# 3D bioprinted human vascular organoid sheets promote functional ischemic repair and exhibit adaptive in vivo remodeling

**DOI:** 10.1186/s12916-026-05256-2

**Published:** 2026-09-25

**Authors:** Xinyu Fu, Li Yan, Zhaosen Chen, Bohan Dou, Dezhi Zhou, Xinyao Zhou, Kengyuan Qu, Che Gao, Peiliang Wang, Fengzhi Zhang, Zihao Zou, Taoxia Wang, Guiying Li, Liliang Ouyang, Jie Na

**Affiliations:** 1 School of Basic Medical Sciences, Tsinghua Medicine, Tsinghua University, 100084 Beijing, China; 2 SXMU-Tsinghua Collaborative Innovation Center for Frontier Medicine, Taiyuan, China; 3 School of Basic Medical Sciences, Shanxi Medical University, Taiyuan, Shanxi Province China; 4 Department of Mechanical Engineering, Tsinghua University, Beijing, China; 5 Beijing Key Laboratory of Intelligent Organ Biofabrication and Regenerative Repair, Tsinghua University, Beijing, China; 6 AngioTherapeutics Ltd, Beijing, China; 7 The First Hospital of Tsinghua University, School of Clinical Medicine, Tsinghua Medicine, Tsinghua University, Beijing, China; 8 Department of General Surgery, Beijing Tsinghua Changgung Hospital, School of Clinical Medicine, Tsinghua University, Beijing, China; 9 Affiliated Hospital of Hebei Engineering University, The Key Laboratory of Basic Research on Blood Purification Application in Hebei Province, Handan, China; 10 State Key Laboratory for Complex, Severe and Rare Diseases, Tsinghua University, Beijing, China

**Keywords:** 3D bioprinting, Blood vessel organoid, Human pluripotent stem cells, Endothelial cells, Smooth muscle cells, Hindlimb ischemia

## Abstract

**Background:**

Ischemic vascular diseases remain a major clinical challenge, creating a need for engineered vascular tissues that can establish functional vascular networks and promote durable tissue repair. Conventional vascular organoids offer limited control over cellular composition and spatial organization, while the fate and adaptive remodeling of graft-derived human vascular cells after transplantation remain poorly understood.

**Methods:**

Human vascular organoid sheets (hVOS) were constructed by extrusion-based three-dimensional (3D) bioprinting of human pluripotent stem cell (hPSC)-derived endothelial cells (ECs) and smooth muscle cells (SMCs) at a defined ratio within a gelatin methacryloyl (GelMA)-based bioink under chemically defined conditions. Vascular organization and cellular states were characterized using functional assays, immunofluorescence imaging, and single-cell RNA sequencing (scRNA-seq). Therapeutic efficacy and graft remodeling were evaluated in a murine hindlimb ischemia model using laser speckle perfusion imaging, histological analysis, intravital two-photon imaging, and scRNA-seq of recovered graft-derived human cells.

**Results:**

Co-bioprinting ECs and SMCs accelerated vascular network formation and generated stable, interconnected vascular structures that underwent progressive maturation during culture. scRNA-seq identified diverse vascular and stromal populations and revealed transcriptional programs associated with vascular maturation, mechanotransduction, and hypoxic adaptation. Following transplantation, hVOS significantly improved blood perfusion, increased limb salvage, and promoted ischemic tissue repair. Intravital imaging detected circulating dextran within GFP-labeled hVOS-derived vascular structures at days 14 and 28, demonstrating perfusion of graft-derived vascular structures by the host circulation. Post-transplantation scRNA-seq revealed substantial adaptive remodeling of graft-derived ECs toward venous-biased and inflammatory states, accompanied by activation of NF-κB- and stress-associated programs. Transplanted SMCs and fibroblasts also exhibited coordinated transcriptional changes associated with wound healing and extracellular matrix remodeling.

**Conclusions:**

hVOS provide a reproducible and design-flexible 3D-bioprinted vascular tissue platform that enables controlled multicellular organization and formation of prevascularized constructs while supporting vascular integration and ischemic tissue repair in vivo. Single-cell analyses further reveal substantial adaptive remodeling of graft-derived vascular and stromal cells following transplantation. These findings support hVOS as a versatile platform for vascular regenerative medicine and for investigating the in vivo behavior of engineered human vascular tissues.

**Figure:**
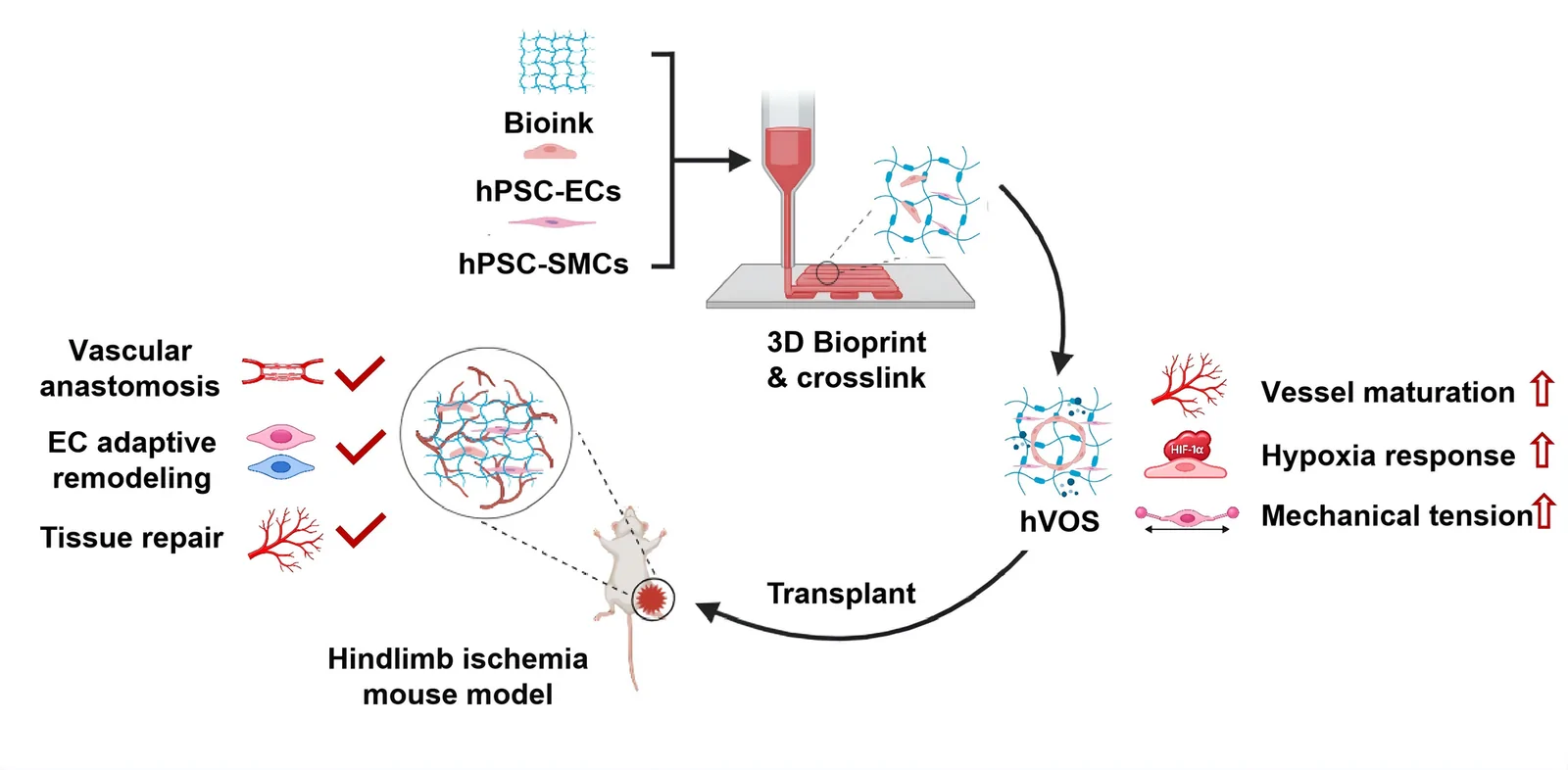

## Highlights

**Reproducible and Customizable 3D Bioprinted hVOS**: hPSC-derived ECs and SMCs self-assembled into stable vascular networks within 3D-bioprinted hVOS, enabling controlled graft fabrication.

**Enhanced Vascular Maturation**: hVOS promoted vascular maturation through coordinated cellular interactions, mechanical support, and hypoxic signaling.

**Therapeutic Efficacy in Hindlimb Ischemia**: Transplantation of 3D-bioprinted hVOS improved blood perfusion and promoted tissue regeneration in a hindlimb ischemia model.

**In Vivo Reprogrammed of EC Function**: Single-cell RNA sequencing revealed that the in vivo microenvironment reshaped EC functions within transplanted hVOS, contributing to vascular repair.

## Background

Ischemic vascular diseases affect approximately 20% of individuals over the age of 50, leading to severe complications such as critical limb ischemia. These conditions are exacerbated in patients with comorbidities, resulting in poor amputation outcomes, elevated mortality rates, and substantial socioeconomic burdens [1–3]. Moreover, many patients are unable to tolerate traditional vascular bypass surgery, emphasizing the urgent need for alternative therapies, particularly in promoting microvascular regeneration and functional restoration [4–6].

Human pluripotent stem cells (hPSCs) offer a promising solution, as they can be efficiently differentiated into the main vascular cell types—endothelial cells (ECs) and smooth muscle cells (SMCs)—and self-organize into vascular networks in vitro [5, 7, 8]. SMCs play a pivotal role in vascular maturation and stability, providing structural support, regulating vessel diameter and tension, and promoting long-term stability of the vascular network [9–11]. However, conventional hPSC-derived vascular organoids typically comprise only capillary-like endothelial networks, lacking mature SMC layers, which limits their structural stability and their ability to fully recapitulate vascular repair in vivo [7]. Moreover, spontaneous organoid formation affords limited control over organoid size, spatial organization, and cellular composition, thereby impeding construct standardization and patient- or disease-specific customization. In contrast to these self-organized vascular organoids, engineered EC/SMC co-transplantation approaches generally deliver vascular cells as suspensions or matrix-embedded mixtures, providing less control over the formation of a preorganized vascular architecture before implantation.

3D bioprinting offers a promising strategy to overcome these challenges and to achieve standardized, customizable fabrication of vascular organoids [12]. Besides, bioinks based on gelatin and its derivatives, such as methacrylated gelatin (GelMA), are particularly well suited for this purpose, as they provide a favorable mechanical and adhesive environment for cells [13], allowing precise control over organoid dimensions, thickness, and spatial architecture [14]. Previous 3D-bioprinted vascular patches have demonstrated the feasibility of combining endothelial and mural cells for ischemic tissue repair. However, many of these systems have relied on primary vascular cells, have been designed primarily as cell-laden therapeutic patches rather than organoid-sheet constructs containing preformed vascular networks, or have not examined the post-transplantation states of graft-derived human cells at single-cell resolution.

Nevertheless, current studies applying hPSC-derived vascular cells or vascular organoids in murine hindlimb ischemia models have mainly focused on demonstrating their ability to improve limb perfusion and promote neovascularization, with therapeutic efficacy largely evaluated by laser Doppler perfusion imaging, vessel density quantification, and conventional histological analysis [15, 16]. In parallel, single-cell RNA sequencing (scRNA-seq) has been widely used in experimental hindlimb ischemia models to systematically dissect the heterogeneity and dynamic remodeling of endogenous cellular populations [17–20]. However, these scRNA-seq studies have predominantly focused on host-derived cells and have rarely directly interrogated the transplanted human grafts within the ischemic limb. Systematic single-cell transcriptomic analyses that track transplanted human cells or vascular constructs in vivo, delineate their survival, lineage and phenotypic remodeling, and elucidate how the ischemic microenvironment reshapes their functional states remain very limited, which substantially constrains our mechanistic understanding of cell- and organoid-based therapies in vivo and limits their rational optimization.

Building on recent progress in vascular organoid engineering, we applied extrusion-based 3D bioprinting to assemble multicellular human vascular organoid sheets (hVOS) composed of hPSC-derived ECs and SMCs at a predefined cellular ratio within GelMA-based bioinks. Unlike conventional self-organized vascular organoids, this approach enabled greater control over cellular composition, construct dimensions, thickness, and geometry. Unlike EC/SMC cell suspensions or matrix-embedded co-transplantation systems, hVOS were implanted as structured sheets containing a preformed vascular network. We further employed a murine hindlimb ischemia model and single-cell transcriptomics to evaluate therapeutic performance and characterize how graft-derived human ECs and SMCs were remodeled after transplantation. Thus, the principal advance of this study lies in the integration of controlled 3D-bioprinted construct design, functional evaluation in ischemic tissue, and post-transplantation single-cell analysis of graft-derived human vascular cells, rather than in any one component alone. Through this combined approach, we aimed to establish a reproducible and design-flexible platform for investigating engineered human vascular tissue remodeling and ischemic repair.

## Methods

### hPSC culture and differentiation

The hiPSCs used in this study were derived from CD34^+^ cells isolated from umbilical cord blood, using the CytoTune™-iPS Reprogramming Kit (Invitrogen) following the manufacturer’s recommended protocol [21]. The H1-GFP cell line used in this study was constructed in our laboratory by ZYX. They were cultured on plates coated with Matrigel (BD Biosciences, distributed by Corning) and maintained in TeSR-E8 medium (STEMCELL Technologies).

The chemically defined medium for EC and SMC differentiation consisted of RPMI 1640 (Thermo), AATS supplement includes 0.5 mg/mL recombinant human serum albumin (OsrHSA HY100C1, Clinical Grade, ORYZOGEN, www.oryzogen.net), 5 μg/mL human Apo-transferrin (T1147, Sigma), 0.2 mg/mL ascorbic acid 2-phosphate magnesium (A8960, Sigma), 5 ng/mL sodium selenite (S5261, Sigma). For mesoderm induction, BMP4 (Peprotech) and CHIR99021 (Tocris) were added at the desired concentrations depending on the cell line. For EC induction, 50 ng/mL VEGF-A (Novoprotein) and 10 ng/mL bFGF (Novoprotein) were added. For SMC differentiation, 10 ng/mL PDGF-BB (Novoprotein) and 5 ng/mL TGFβ1 (Novoprotein) were added. ROCK inhibitor Y27632 (TOCRIS) (5 μM) was added during passaging for 24 h and removed with the medium change.

### Bioink preparation and cell printing

GelMA (SunP Biotech) and Matrigel (BD Biosciences) were used to formulate bioinks. For GelMA bioink, 5% w/v of GelMA containing 0.25% w/v LAP photoinitiator was prepared using RPMI 1640 and passed through a 0.22 μm filter before use. For GelMA-Matrigel (G-M) bioink, 5% w/v of GelMA and 10% w/v Matrigel containing 0.25% w/v LAP photoinitiator was prepared by gentle mixing at 4°C. After mixing with an appropriate number of cells, ECs bioink containing 3 × 10^6^ hPSC-ECs mL^-1^, hVOS bioink containing 2 × 10^6^ hPSC-ECs mL^-1^ and 1 × 10^6^ hPSC-SMCs mL^-1^, HUVECs bioink containing 3 × 10^6^ HUVECs mL^-1^ and HUVECs + hPSC-SMCs bioink containing 2 × 10^6^ HUVECs mL^-1^ and 1× 10^6^ hPSC-SMCs mL^-1^ were prepared.

Cell Maker 4 bioprinter (SunP Biotech) was used to fabricate blood vessel organoid sheets. The cell-laden bioinks were loaded into a 3 mL syringe and then printed into a sheet structure (8 × 8 × 0.4 mm) at 19 °C. The 8 mm × 8 mm × 0.4 mm sheet geometry was selected to provide broad coverage of the ischemic region, facilitate surgical handling and local retention, increase the tissue-contact area, and maintain a relatively short diffusion distance for oxygen and nutrients during the early post-transplantation period. The printed structures were then exposed to 405 nm light (30 mW cm^−2^, 20 s) for crosslinking. The culture medium was refreshed every day.

### Measurement of mechanical and rheological properties

Rheological properties were measured using an Anton Paar MCR 302 rotational rheometer in controlled-rate mode. A plate–plate geometry (25 mm diameter and 0.1 mm gap) was used for the measurements on this instrument. Steady shear sweeps were used to investigate the rheological properties of the material by recording the viscosity at increasing shear rates from 0.01 to 10 (1/s) at 19°C. Oscillatory frequency sweeps were conducted with a linearly ramped temperature from 40 to 4°C at a frequency of 1.5 Hz and strain of 1%. The complex viscosity of hydrogels was measured as a function of temperature decrease from 40 to 4 °C in oscillatory frequency sweep mode.

### Cryogenic scanning electron microscopy (Cryo-SEM) analysis of Hydrogel

Hydrogel samples were initially immersed in a nitrogen slurry for vitrification. The frozen samples were then transferred to a preparation chamber, where they were subjected to brittle fracture using a cryo-blade at -150 °C. This process exposed the internal cross-sectional structure of the sample. The fractured samples were subsequently placed in a vacuum environment at -90 °C for a 5-minute sublimation treatment. A thin conductive platinum layer (approximately 5 nm thick) was then deposited onto the sample surface using an ion sputter coater under the following parameters: 5 mA for 60 s. Finally, the treated samples were transferred to a Cryo-SEM chamber. Imaging was performed using a FEI Helios NanoLab G3 UC field-emission SEM equipped with a cryo-stage. All low-temperature procedures were carried out under the control of a Gatan Alto 2500 cryo-transmission system.

### Cell viability analysis

Cell viability was assessed using the Live/Dead Cell Viability Kit (KEYGEN BIOTECH) according to the manufacturer’s protocol. Briefly, the medium was removed, and then the structures were washed three times with PBS. Subsequently, a PBS solution containing 2 mM Calcein-AM and 8 mM propidium iodide was added to stain the hydrogel structures. Structures were immersed in the staining solution at room temperature in the dark for 10 mins, then washed three times with PBS. Images were acquired on a fluorescence microscope (NIKON). Live and dead cells in each sample were counted in three randomly selected fields.

### CCK-8 cell proliferation assay

Cell proliferation was assessed using a Cell Counting Kit-8 (40203ES60, YEASEN) according to the manufacturer’s instructions. Briefly, CCK-8 reagent was added to each well at 10% of the culture medium volume. The cells were subsequently incubated at 37°C in a humidified atmosphere containing 5% CO₂ for 4 h. Absorbance at 450 nm was measured using a microplate reader. Cell-free wells containing culture medium and CCK-8 reagent were used as blanks, and the blank-corrected absorbance values were used to evaluate relative cell proliferation.

### Hydrogel lysis and cell collection

The GelMA hydrogel structure was retrieved, and the medium was removed from the culture wells. A working solution of GelMA lysate (SunP Biotech) was then added to fully immerse the structure. The scaffolds were placed in a 37 °C incubator for aseptic lysis, with the lysis process observed under a microscope every 10 minutes. Following sufficient lysis, the supernatant was discarded after centrifugation, and the desired cells were collected after 1-2 washes with PBS.

### Immunostaining and image analysis

For whole-mount immunostaining of 3D printed structures, they were fixed with 4% paraformaldehyde, permeabilized in 0.2% Triton X-100 (Sigma), blocked in 5% bovine serum albumin (Solarbio), and then incubated with primary antibodies at 4 °C overnight. Antibodies used in this study included hCD31 (1:100, Santa Cruz, SC81158), hCD31 (1:400, Abcam, ab9498), VE-cadherin (1:200, R&D, AF938), SM22a (1:400, Abcam, ab14106), hKu80 (1:200, Cell Signaling Technology, #2180), CD68 (1:200, Thermo, 14-0688-82), CD31 (1:200, Immunoway, YM8207), HIF1A (1:200, Novus, NB100-105SS), CD34 (1:500, Abcam, ab81289), Anti-GFP (1:200, Abcam, ab1218). Then they were detected by DyLight 488-conjugated (EarthOx), DyLight 549-conjugated (EarthOx), CoraLite 647-conjugated (Proteintech), FITC-conjugated (Proteintech) and Cy3-conjugated (Proteintech) secondary antibodies. Nuclei were stained with DAPI (Solarbio). The Nikon Ti–U fluorescence microscope, A1R confocal microscope and SpinSR confocal microscope were used for image acquisition. The images were processed using Imaris 10.2 and analyzed using the Filaments function.

For immunofluorescence staining of cryosections, the printed constructs were initially fixed in 4% paraformaldehyde and then dehydrated in 30% sucrose. The structures were then embedded in the optimal cutting temperature (OCT) compound and sectioned using a Leica CM3050S cryostat to obtain 40 µm thickness slices. After drying at 50 °C for 1 hour, immunofluorescence staining was performed. The reagents and procedural steps were identical to those used for whole-mount immunostaining of 3D printed structures. The images were processed with Fiji ImageJ.

### Flow cytometry analysis

Cells were dissociated into single cells using Accutase (Millipore) and resuspended in a FACS washing buffer (PBS with 5% fetal calf serum (FCS) and 2.5 mM EDTA). The following antibodies used in this study are FITC-conjugated anti-CD31 (1:100, BD Biosciences, 555445), Alexa Fluor®647-conjugated anti-CD144 (1:100, BD Biosciences, 561567), FITC anti-hCD31 (1:100,BD Biosciences, 303104), PE-CD73/NT5E (1:100, BioLegend, 344003), APC-Cy7-CD184/CXCR4 (1:100, BioLegend, 306527). The dissociated cell suspension was incubated with the fluorophore-labeled primary antibody at room temperature for 20 min. The corresponding mouse IgG (1:100, BioLegend) was used as an isotype control. Data were collected with a FACSCaliber flow cytometer (BD) and analyzed using FlowJo (version 10.0.7).

### hPSC-EC and hPSC-SMC functional test

The biological function of hPSC-ECs was assessed via vascular tube formation assay. Specifically, 1 × 10^5^ cells were plated into one well of 24-well plates coated with Matrigel (BD) and incubated at 37 °C for 12 h. To test the contractile ability of differentiated SMCs, SMCs were treated with 30 μM carbachol. After incubation for 30 min, the cell-surface area was measured.

### Single-cell RNA-sequencing and data analysis

The bioprinted hVOS were lysed with the working solution of GelMA lysate (SunP Biotech) following the instructions. After sufficient lysis, the supernatant was discarded after centrifugation, and released cells were collected and washed with PBS. Single-cell RNA-Sequencing (scRNA-seq) was performed following SeekOne® Digital Droplet instrument instruction. Raw data were mapped to a GRCh38 and Rnor_6.0 mixed reference genome using STARsolo pipeline (version:2.7.8a), which performed reads mapping, cell demultiplexing, UMI counting, and initial cell filtering. The generated UMI matrix was then analyzed using Suerat (V4). Cells with lower than 2000 UMIs and 1000 genes were filtered out. Cell clustering and UMAP embedding were performed using the top 15 principal components. The resolution in the FindClusters() function was set to 0.1, Cell types were annotated based on established cell type markers. To compare the single-cell transcriptome of ECs and SMCs in 2D culture with that from 3D bioprinted hVOS, we used a previously published hPSC-derived EC and SMC scRNA-seq dataset (GSE166462) [22]. The EC or SMC populations from two datasets were combined, and differential expression analysis was performed using the FindMarkers() function with the default Wilcoxon Rank Sum test. The UMAP plots of selective genes were shown based on their known function in vascular development. Cell-cell interaction and receptor-ligand analyses were performed using the curated ligand-receptor interaction database from the CellChat package (version 1.5).

### In vivo hindlimb ischemia (hli) model and graft implantation in mice

All animal procedures were carried out in compliance with the guidelines approved by the Institutional Review Board of the Laboratory Animal Research Center at Tsinghua University. Male BALB/c nude mice (aged 8-10 weeks, weighing 20-25 g) were anesthetized with 2% isoflurane administered at a flow rate of 1 L/min. After creating a skin incision, the femoral neurovascular bundle was visualized under an anatomical microscope. The left hindlimb femoral artery was meticulously isolated from the femoral vein and nerve. The hindlimb ischemia was induced by ligating the proximal end of the femoral artery and its branches using 5-0 silk sutures, as detailed in [23]. In this HLI model, perfusion of the affected limb was evaluated relative to the unaffected limb. After vascular resection, 3D bioprinted hVOS were applied to the ligated and severed femoral artery region, and the skin was closed using 5-0 silk sutures. The transplant sites were regularly checked for signs of infection or scarring.

Mice were divided into four groups: sham (only creating a skin incision and then suture), ischemia-only (HLI), ischemia with G-M bioink (no cells), and ischemia with 3D bioprinted hVOS (containing SMC and EC). After surgery, the mice’s activities were monitored on days 7, 14, and 28. Microvascular blood flow in the hindlimbs was measured using a laser speckle flowgraphy (MoorFLPI-2, Moor Instruments, Axminster, UK). Perfusion of the operated lower limb after surgery was evaluated relative to the contralateral side. At the end of the experiment, all mice were euthanized via carbon dioxide inhalation. The implants and muscle tissues were collected and cryopreserved at -80 °C. Blood flow data from the ischemic and normal hindlimbs of each mouse were compared and presented as percentages.Gross outcomes of the ischemic hindlimbs were evaluated and classified into three mutually exclusive categories: limb salvage, defined as complete preservation of the ischemic hindlimb without visible foot necrosis; foot necrosis, defined as visible blackening, dry gangrene, or tissue necrosis involving one or more toes or the foot without loss of the entire limb; and limb loss, defined as spontaneous autoamputation of the ischemic hindlimb resulting from extensive ischemic necrosis.

### Hematoxylin and eosin (H&E) staining

Sections were stained with hematoxylin for 3-5 minutes, then incubated in acid alcohol and alkaline water. Subsequently, sections were counterstained with eosin for 1-2 minutes, dehydrated through graded ethanol, cleared in xylene, and mounted with a neutral resin.

### Intravital two-photon imaging

Mice were anesthetized by intraperitoneal injection of tribromoethanol (150 μL per 10 g body weight). After adequate anesthesia was confirmed, the skeletal muscle of the ischemic hindlimb was surgically exposed. The nonabsorbable suture used for femoral artery ligation during establishment of the hindlimb ischemia model served as an anatomical landmark to identify the region surrounding the transplantation site and guide precise imaging. The mice were subsequently positioned and immobilized using the specimen stabilization module of an upright two-photon microscope system (FVMPE-RS, Evident). Five minutes before imaging, each mouse received a tail-vein injection of 100 μL Texas Red–labeled dextran solution (Texas Red–dextran, D1830, Thermo Fisher Scientific; 70 kDa; 1.67 mg in 100 μL PBS) to label the circulating plasma and visualize functionally perfused vascular lumens. Intravital two-photon imaging was then performed in the transplantation region to assess perfusion of GFP⁺ hVOS-derived vascular structures by the host circulation.

### Graft digestion and cell collection

Reagents were prepared as follows: the wash medium (WM) consisted of RPMI 1640 with 10% fetal bovine serum (FBS) and 1× penicillin/streptomycin, kept at 4–8°C throughout the experiment. Collagenase II was prepared by dissolving collagenase II powder in RPMI 1640 to a final concentration of 1000 U/ml, filtered through a 0.22 μm filter for sterilization, aliquoted into 1 ml tubes, and stored at -20°C. Dispase solution was prepared in a similar manner, with a final concentration of 11 U/ml, sterilized, aliquoted, and stored at -20°C. Prior to use, both solutions were thawed and centrifuged at >10,000 rpm for 1 minute to precipitate protein aggregates, and the supernatant was used for digestion.

Hind limb muscle tissues (1 cm × 1 cm × 1 cm) were collected under a microscope, washed thoroughly with PBS to remove blood and debris, and transferred into 1.5 ml tubes on ice. The tissue was minced with surgical scissors for 1-2 minutes until a homogeneous slurry was obtained. Then 0.5 ml collagenase II and 0.5 ml dispase solutions were added to the minced tissue, gently mixed, and incubated on a 37°C, 220 rpm shaker for 30 minutes. After digestion, the suspension was transferred to a new 15 ml centrifuge tube, and 1 ml of 0.25% trypsin-EDTA solution was added. The mixture was incubated for an additional 5 minutes. The digested suspension was then filtered through a 70 μm cell strainer into a 50 ml tube placed on ice. Remaining tissue fragments on the strainer were gently crushed using the rubber stopper of a 1 ml syringe to facilitate more cell passage. The strainer was rinsed with 1-2 ml wash medium, and the rinse was added to the 50 ml tube. The cell suspension was centrifuged at 500 g for 3 minutes, and the supernatant was discarded. The cell pellet was resuspended and used for flow cytometry and scRNA-seq experiments.

### Statistics

Statistical analyses were performed using GraphPad Prism 7.0. Data are presented as mean ± SD unless otherwise specified. For comparisons between two groups, two-tailed unpaired t-tests were used, and one-way ANOVA was applied for analyses involving three groups. Statistical significance was defined as p < 0.05. For image-based quantification, parameters obtained from multiple samples are reported as mean ± SEM. In animal studies, n = 6 mice per group, with more than 10 images analyzed per mouse, and results are presented as mean ± SEM.

## Results

### 3D bioprinting generated hVOS from hPSC-derived ECs and SMCs

**Fig. 1:**
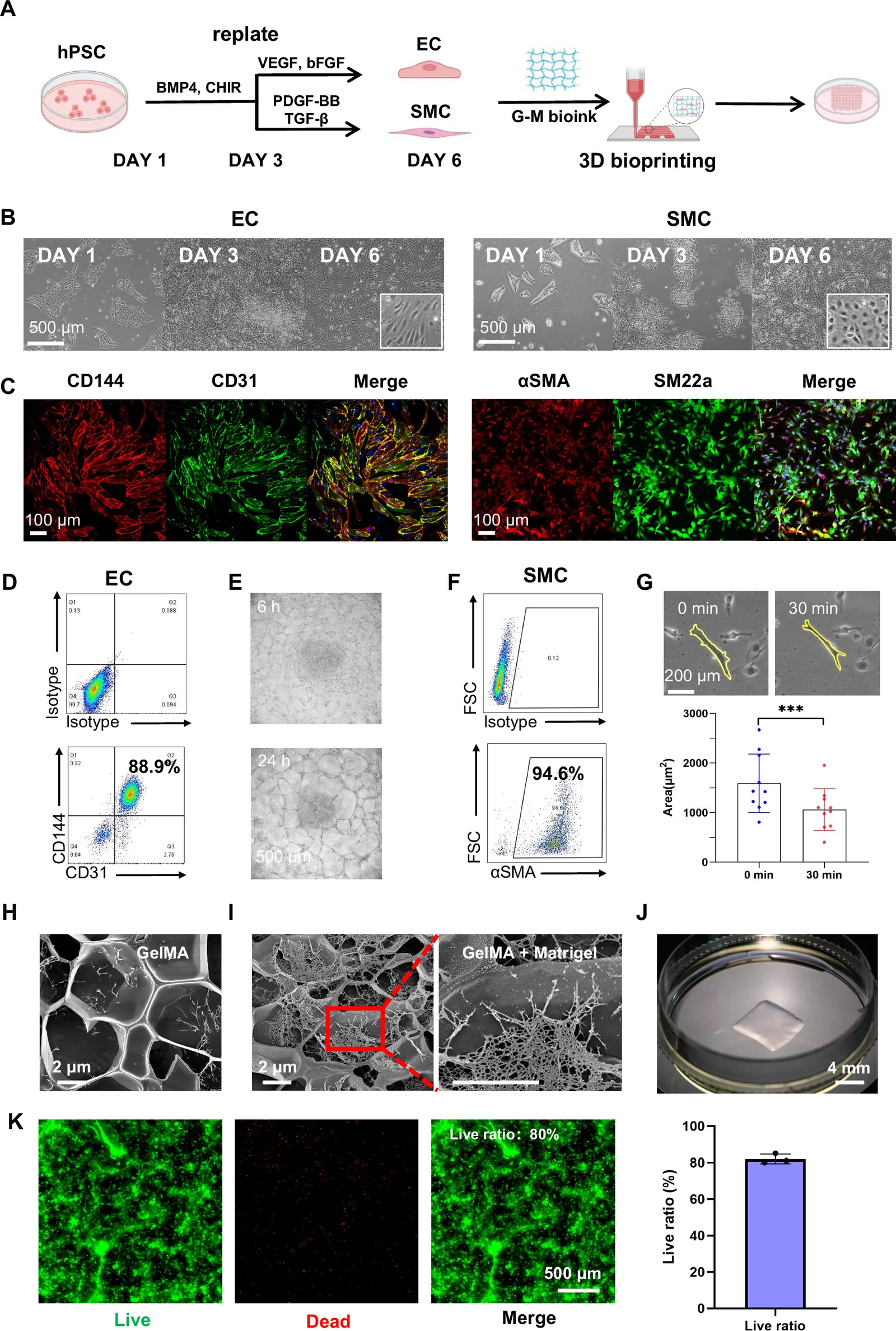
Generation of hVOS through 3D bioprinting. **A** Schematic diagram of the ECs and SMCs differentiation and 3D bioprinting process. **B** Bright field images of different days of induction from hPSCs to ECs and SMCs. **C **Representative immunofluorescence staining of hPSCs-ECs and hPSCs-SMCs. **D** Flow cytometry analysis results of hPSCs-SMCs. **E** Tube formation assay of hPSC-ECs. **F** Flow cytometry analysis results of hPSCs-SMCs. **G** TOP: Phase-contrast images of hPSCs-SMCs before (0 min) and after (30 min) 30 μM carbachol treatment. BOTTOM: Related quantitative analysis of cell-surface area changes upon carbachol treatment (n = 10 cells, ***p < 0.001, two-tailed unpaired Student’s t-test). **H** Cyro-SEM images of 5% GelMA Hydrogel. **I** Cyro-SEM images of 5% GelMA+10% Matrigel Hydrogel. **J** Bioprinting structures with GM hydrogel. **K** LEFT: Cell viability of hVOS cultured for 3 days tested by Calcein AM/PI staining. RIGHT: Related quantitative analysis of cell viability of bioprinting structures (*n* = 3 biological replicates)

Previously, we established a chemically defined, simple, and efficient protocol for differentiating endothelial cells (ECs) and smooth muscle cells (SMCs) from hPSCs (Fig. 1A) [24]. Briefly, hPSCs were induced toward the mesodermal lineage by three days of BMP4 and CHIR99021 treatment in AATS medium. The resulting mesodermal cells were then passaged and exposed to VEGF and bFGF for three days to generate ECs, whereas treatment with PDGF-BB and TGFβ over the same period efficiently directed differentiation toward SMCs. The differentiated populations displayed characteristic EC and SMC morphologies (Fig. 1B), and immunofluorescence and flow cytometry confirmed robust expression of lineage-specific markers (Fig. 1C, D, F). Functional assays further demonstrated that ECs formed tubular networks (Fig. 1E) and that SMCs exhibited contractile responsiveness, as evidenced by a marked reduction in cell surface area after 30 minutes of stimulation with 30 μM carbachol (Fig. 1G). These well-characterized ECs and SMCs provided a reliable cellular source for subsequent 3D bioprinting.

To enable the bioprinting of human vascular organoid sheets (hVOS) from these differentiated cells, we next focused on preparing and optimizing suitable bioinks. Methacrylated gelatin (GelMA), a widely used hydrogel for 3D cell culture and bioprinting [25], was combined with Matrigel, which recapitulates key biochemical cues of the native basement membrane and supports vascular cell adhesion, proliferation, and differentiation [26]. Based on these properties, we formulated a GelMA–Matrigel (G-M) bioink by mixing 10% (w/v) Matrigel with 5% GelMA. Rheological characterization revealed that the complex viscosity of both GelMA and G-M bioinks increased as temperature decreased, with Matrigel addition modestly increasing viscosity (Additional file 1: Figure S1A). Both formulations exhibited pronounced shear-thinning behavior across shear rates of 0.01–100 s⁻¹, consistent with requirements for extrusion-based bioprinting (Additional file 1: Figure S1B, C). Temperature-dependent oscillatory rheology further showed that the G-M bioink maintained a more stable gel state above 30°C compared with GelMA alone (Additional file 1: Figure S1D). Cryo-SEM imaging confirmed that the G-M hydrogel formed a porous GelMA framework interlaced with Matrigel proteins (Fig. 1I), in contrast to the simpler structure of 5% GelMA (Fig. 1H), thereby creating a supportive network for vascular cell integration.

To further evaluate the biological performance of the cell-laden G-M bioink, hPSC encapsulation within G-M constructs resulted in high cell viability after one day of bioprinting (Additional file 1: Figure S1E), and CCK-8 analysis demonstrated sustained cell proliferation during culture, with enhanced proliferation compared with 5% GelMA at later time points (Additional file 1: Figure S1F). When hPSC-derived ECs and SMCs were incorporated into the G-M bioink, bright-field imaging showed progressive cell migration and vascular-like structure formation during culture (Additional file 1: Figure S1G).

Because EC:SMC composition influences vascular organization, we further compared EC:SMC ratios of 3:1, 2:1, and 1:1. Among these conditions, the 2:1 ratio generated more continuous CD31-positive vascular networks with increased vessel area, length, and junction numbers (Additional file 1: Figure S2A, B), and was therefore selected for subsequent hVOS fabrication.

Using this optimized G-M bioink, we established a reproducible workflow for fabricating hPSC-derived vascular organoid sheets (hVOS) measuring 8 mm × 8 mm × 0.4 mm, tailored to the size of ischemic mouse hindlimbs (Fig. 1J). Live/dead staining after 3 days of culture (Fig. 1K) demonstrated high cell viability, underscoring both the excellent biocompatibility of the G-M formulation and the fidelity of the bioprinting process.

### hVOS supported the assembly of structurally robust vascular networks

**Fig. 2:**
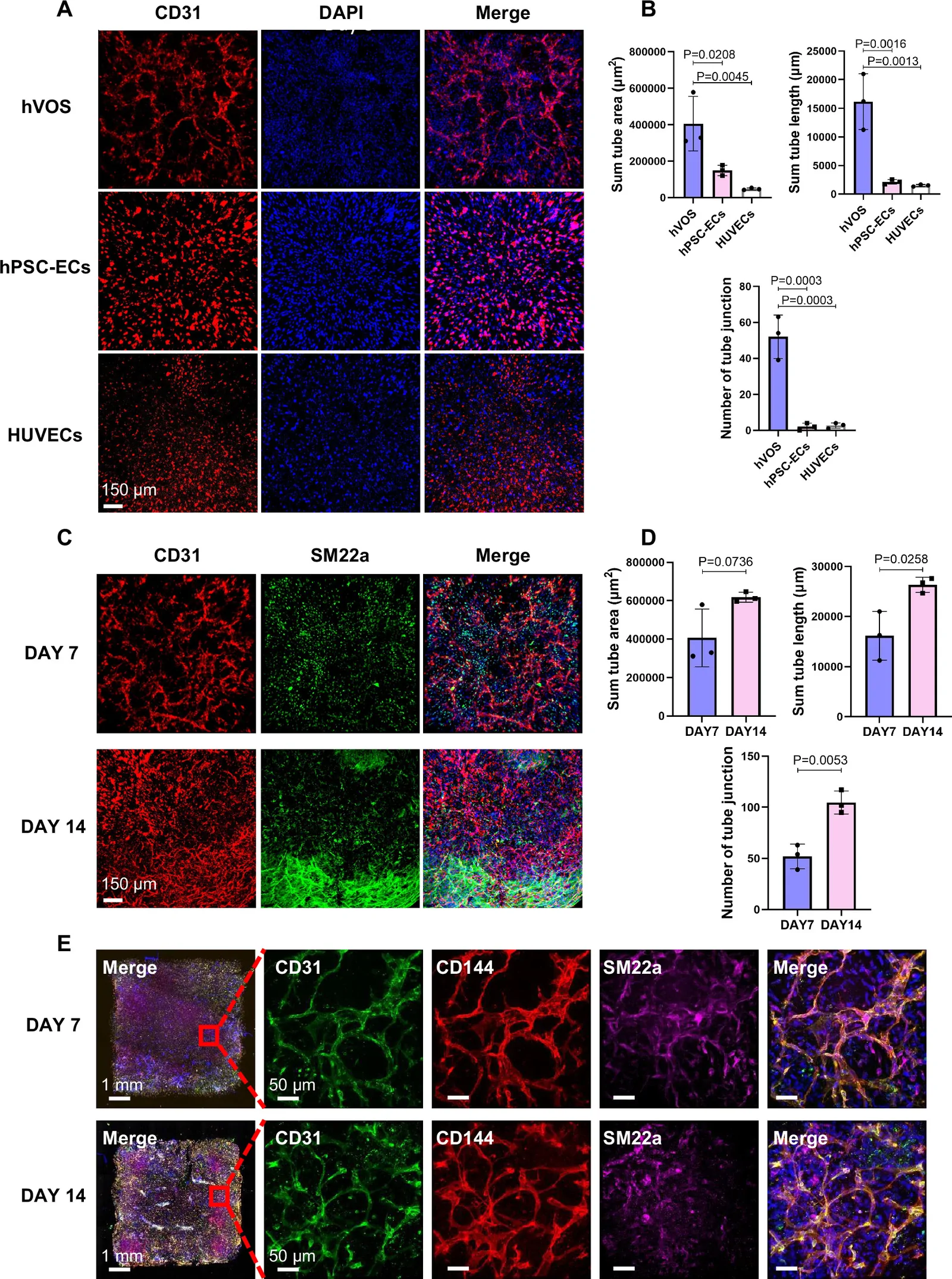
3D bioprinting promoted the formation of a more robust vascular network. **A** Representative immunofluorescence staining of 3D bioprinted hVOS, hPSC-ECs, HUVECs on 7 days. **B** Related quantitative analysis of the sum tube area, the sum tube length, and the number of tube junction (*n* = 3 biological replicates, one-way ANOVA test). **C **Representative immunofluorescence images showing vascular networks formed by ECs and SMCs in hVOS after 7 and 14 days of culture. **D** Related quantitative analysis of sum tube area, sum vessel length, and number of vessel junction (*n* = 3 biological replicates, two-tailed unpaired Student’s t-test). **E** Representative high-resolution immunofluorescence images of hVOS at day 7 and day 14

To visualize and quantify the vascular network in bioprinted hVOS, immunofluorescence analysis was performed, confirming that 3D bioprinted hVOS developed more continuous and mature vascular networks compared with control groups. By day 7, hVOS displayed thicker, well-connected tubular structures with strong CD31 expression (Fig. 2A), whereas constructs composed of hPSC-derived ECs alone, HUVECs alone, or HUVECs combined with hPSC-derived SMCs showed only sparse, discontinuous CD31-positive segments (Fig. 2A, Additional file 1: Figure S2C). Quantitative metrics showed significantly increased tube junctions, tube area, and tube length in hVOS compared with all other groups (Fig. 2B, Additional file 1: Figure S2D).

Collectively, these findings demonstrate that hPSC-derived ECs and SMCs rapidly self-organize after bioprinting to form stable vascular networks that undergo progressive structural maturation within the 3D construct.

### ScRNA-seq analysis revealed the cellular composition and molecular characteristics of cell populations within hVOS

**Fig. 3:**
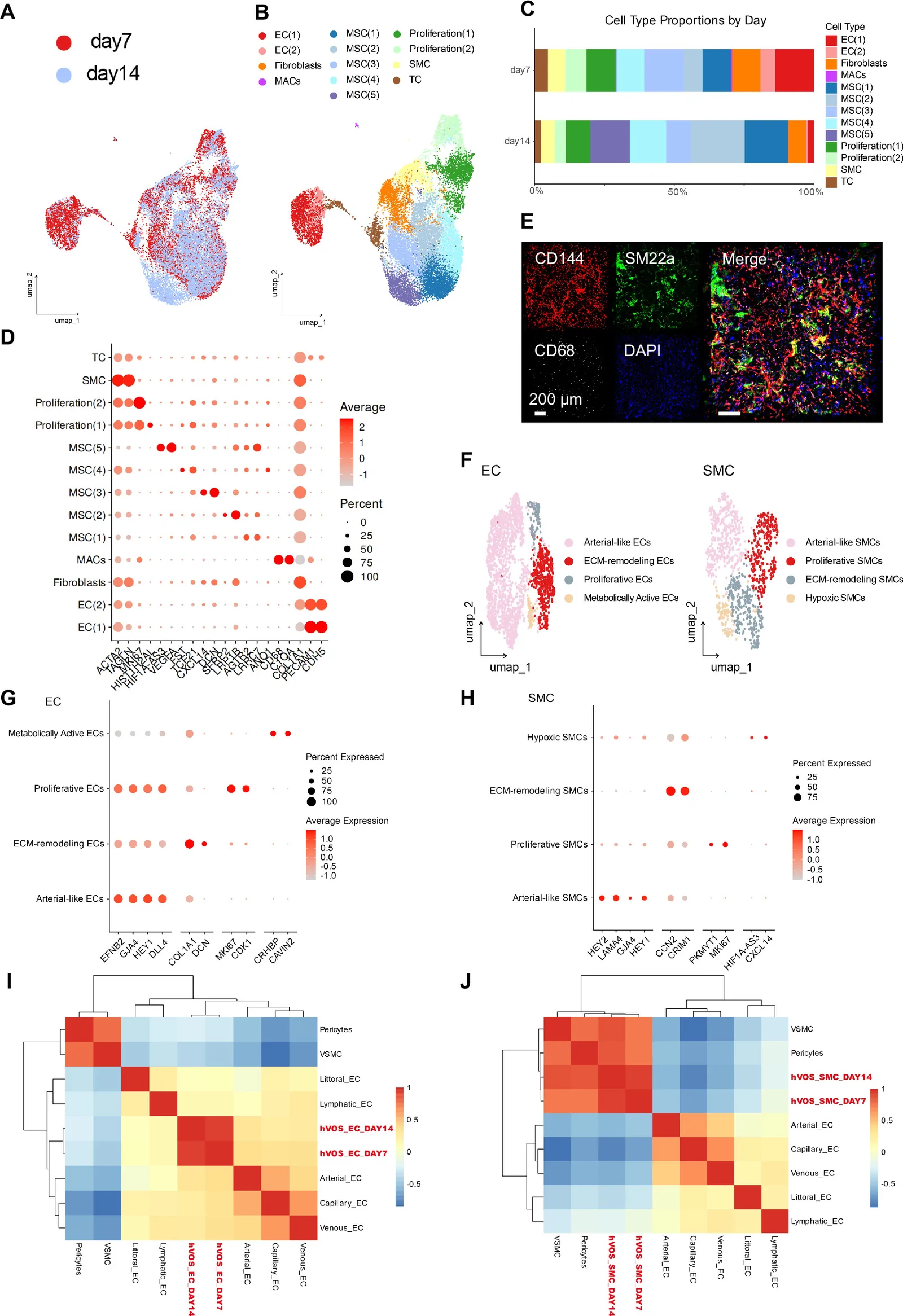
ScRNA-seq analysis of hVOS. **A** UMAP visualization of the scRNA-seq dataset from the 3D bioprinted hVOS grouped by day. **B** UMAP visualization of the scRNA-seq dataset from the 3D bioprinted hVOS grouped cell type. **C** Cell type proportion by day. **D** Dot plot showing expression of cluster marker genes. Dot color represents the scaled average expression level; dot size indicates the percentage of cells within each cluster expressing the gene. **E** Representative immunofluorescence results of CD144, CD68 and SM22a on day 7 hVOS. **F** UMAP visualization showing the subtypes of ECs and SMCs in 3D bioprinted hVOS. **G** Dot plot showing expression of marker genes for EC subtype clusters. Dot color represents the scaled average expression level; dot size indicates the percentage of cells within each cluster expressing the gene. **H** Dot plot showing expression of marker genes for SMC subtype clusters. Dot color represents the scaled average expression level; dot size indicates the percentage of cells within each cluster expressing the gene. **I** Heatmap based on correlation analysis of the scRNA-seq dataset from the 3D-bioprinted hVOS ECs with vivo vascular cells. **I** Heatmap based on correlation analysis of the scRNA-seq dataset from the 3D-bioprinted hVOS SMCs with vivo vascular cells

To delineate the cellular composition and intrinsic molecular features of hVOS, we performed single-cell RNA sequencing (scRNA-seq) on day-7 and day-14 cultures and conducted an integrated analysis. Across both time points, seven major cell types were identified: ECs, SMCs, fibroblasts, mesenchymal stromal cells (MSCs), proliferative-state cells, transition-state cells (TCs), and a minor population of macrophages (Fig. 3A–C). The corresponding Gene ontology (GO) enrichment analysis showed that ECs were enriched in “blood vessel endothelial cell migration” and “vasculogenesis” pathways associated with angiogenesis, while SMCs were enriched in “regulation of cell-substrate adhesion” and “contractile actin filament bundle assembly”, which are essential for smooth muscle contractile function and vascular formation, and fibroblasts were enriched in “extracellular matrix organization” and “collagen fibril organization”, both of which are closely related to angiogenesis and vascular stabilization (Additional file 1: Figure S3A-C). Based on the marker gene expression, the ECs can be separated into two subgroups: EC (1) with high *PECAM1* and *CDH5* expression, and EC (2) with relatively lower expression of these genes; SMCs expressed *ACTA2* and *TAGLN* at high levels; fibroblasts expressed *COL1A1*. MSCs are subdivided into five subsets: subset 1 (high *FST*, *ANO1*; features consistent with ECM regulation), subset 2 (high *AGTR2*, *LRRC7*; pericyte-like features), subset 3 (high *SFRP2*, *LRP1B*; potentially involved in cell signaling regulation and transduction), subset 4 (high *CXCL14*, *DCN*; fibroblast-like features), and subset 5 (high *HIF1A-AS3*, *VEGFA*; suggestive of hypoxia response and pro-angiogenic functions). Proliferative cells comprised two types: one with high *HIST1H2AL* and the other with high *MKI67* (Fig. 3D). Notably, despite the absence of exogenous factors that induce macrophage differentiation, macrophages arose spontaneously by day 7, expressing both canonical macrophage markers (Fig. 3D); GO enrichment indicated upregulation of immune-related pathways, including the “immune response-activating signaling pathway” and “phagocytosis” (Additional file 1: Figure S3D). Immunofluorescence corroborated the scRNA-seq findings (Fig. 3E). We speculate that the porous network of the G-M bioink may provide a permissive microenvironment for their emergence [27].

Classification guided by marker genes associated with physiological states further resolved the EC and SMC compartments into specialized subpopulations. ECs segregated into four distinct states: Arterial-like ECs with high *GJA4* and *HEY1* expression, ECM-remodeling ECs enriched for *COL1A1* and *DCN*, Proliferative ECs marked by *MKI67* and *CDK1*, and Metabolically Active ECs expressing *CRHBP* and *CAVIN2* (Fig. 3G, F). SMCs similarly resolved into Arterial-like SMCs (*HEY2* and *LAMA4*), ECM-remodeling SMCs (*CCN2* and *CRIM1*), Proliferative SMCs (*MKI67*), and Hypoxic SMCs characterized by *HIF1A* (Fig. 3F, 

H). These findings demonstrated that hVOS harbors multiple vascular cell states spanning diverse maturation and functional programs. Integrative comparison with public single-cell atlases of human vasculature [28] further revealed strong transcriptional concordance between hVOS-derived ECs/SMCs and their in vivo counterparts, indicating that hVOS faithfully recapitulates key molecular features of native vessels (Fig. 3I, J; Additional file 1: Figure S3E, F).

In summary, hVOS contains diverse vascular-related cell populations and can spontaneously generate immune components; its cellular and molecular profiles closely parallel those of in vivo vasculature, supporting hVOS as an effective model for studying human vascular development, maturation, and repair mechanisms.

### Cell type communication analysis uncovered extensive interactions and activated signaling networks in hVOS

**Fig. 4:**
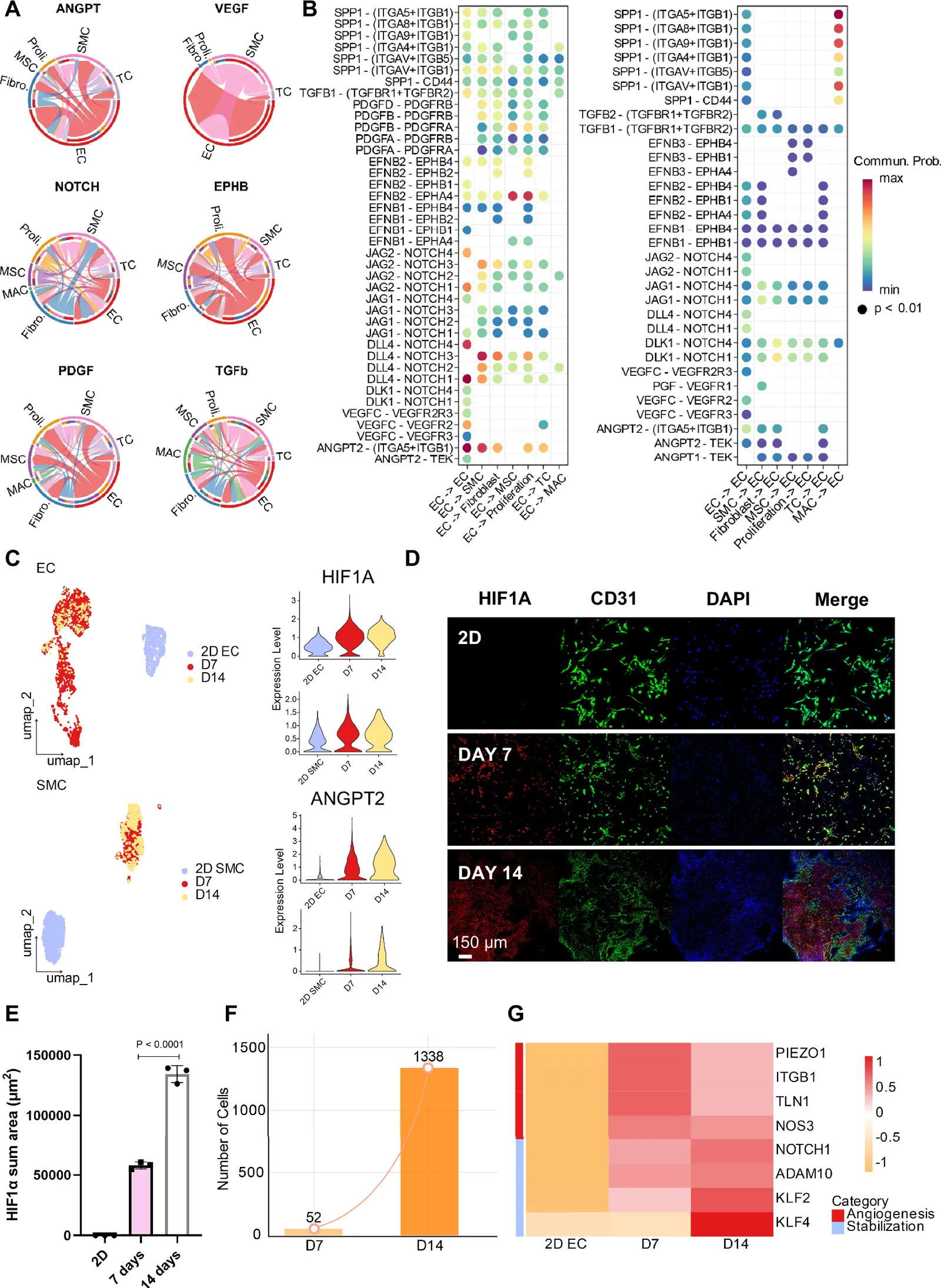
Cell-cell interaction and signaling pathway analysis of different cell types in hVOS. **A** Intercellular communication patterns among different signaling pathways within the hVOS. **B** Ligand–receptor communication in angiogenesis and vessel maturation pathways in hVOS. Right: significant signaling interactions where ligands are secreted by ECs and receptors are expressed by other cell types. Left: significant ligand–receptor interactions where ligands are secreted by other cell types and receptors are expressed by ECs. **C** Left: UMAP visualization of the scRNA-seq dataset from the 3D-bioprinted hVOS ECs and SMCs with 2D cells. Right: Violin plot visualization of HIF1A and ANGPT2 gene expression. **D** Representative immunofluorescence staining of the HIF1A and CD31. **E** Related quantitative analysis of HIF1A channel sum area (*n* = 3 imaging fields, two-tailed unpaired Student’s t-test). **F** MSC (hypoxia) cell population dynamics from 7days to 14days. **G** Heatmap of EC mechanosensing and angiogenesis genes expressed programs

To clarify cell–cell communication programs that support angiogenesis and subsequent vascular stabilization in hVOS, we applied CellChat [29] with ECs as the reference population and systematically surveyed ligand–receptor interactions in which ECs acted as signal senders or receivers, including potential EC autocrine loops. This analysis revealed broad and robust crosstalk between ECs and other cell types, most prominently with SMCs, fibroblasts, and MSCs, as well as additional interactions with macrophages. Pathway-level signals were enriched for established angiogenesis/maturation axes, including VEGF, ANGPT, NOTCH, EPHB, PDGF, and TGFβ (Fig. 4A).

In sender mode, EC-derived ANGPT2 was predicted to interact with the integrin complex ITGA5–ITGB1, consistent with enhanced EC motility and sprouting [30]. EC-expressed JAG and DLL4 were linked to NOTCH receptors on neighboring ECs, supporting Notch-dependent vascular patterning [31], and VEGFC–VEGFR interactions were also enriched, consistent with VEGF-driven EC proliferation, migration, and lumen formation during early angiogenesis [32]. As vascular networks shifted toward maturation, EC communication with perivascular/matrix populations increased, with prominent PDGF–PDGFR and JAG/DLL4–NOTCH signaling associated with recruitment of mural/mesenchymal cells (including SMCs and fibroblasts) and vessel wall reinforcement [33, 34]. EFNB–EPHA interactions were also enriched, suggesting ephrin–Eph signaling acting alongside PDGF to coordinate mesenchymal recruitment and promote stabilization [35]. Despite their low abundance, macrophages were predicted to signal via an SPP1–(ITGA5 + ITGB1) module, consistent with a paracrine contribution to vascular assembly and nascent vessel maturation [36, 37] (Fig. 4B).

The results revealed broad and strong signaling interactions between ECs and other cell types, most prominently with SMCs, fibroblasts, and MSCs, as well as detectable crosstalk with macrophages. Pathway-level analyses of angiogenesis/maturation-related axes (VEGF, ANGPT, NOTCH, EPHB, PDGF, and TGFβ) showed multiple parallel signaling routes between ECs and these matrix/wall cell types, consistent with established roles of VEGF-NOTCH in tip–stalk specification and PDGF in pericyte/SMC recruitment and vessel maturation [38, 39].

Given that the 3D construct provides sustained mechanical cues and may generate a relatively hypoxic microenvironment, we examined whether hypoxia-responsive and mechanotransduction-related transcriptional programs were associated with vascular organization and maturation in hVOS. Hypoxia is known to activate HIF-related signaling and induce pro-angiogenic gene expression [40]; Comparative transcriptomic analysis between hVOS and 2D cultures revealed increased expression of hypoxia-associated genes, including HIF1A and ANGPT2, in hVOS (Fig. 4C). In addition, the MSC (5) population, characterized by expression of hypoxia-related genes such as HIF1A-AS3 and VEGFA, increased in relative abundance from day 7 to day 14 (Fig. 4F). Consistently, immunofluorescence analysis showed increased HIF1A-positive signals in 3D cultures over time compared with 2D conditions (Fig. 4D, E). These findings suggest that 3D microenvironment is associated with enhanced hypoxia-responsive and pro-angiogenic transcriptional features. With respect to mechanotransduction, endothelial responses to flow and shear in vivo typically proceed in two stages: (i) activation of the mechanosensitive ion channel PIEZO1, triggering Ca²⁺ signals and transcriptional programs that facilitate nascent vessel formation [41, 42]; and (ii) sustained induction of the flow-responsive transcription factor KLF2 (and associated programs), which promotes anti-inflammatory, antithrombotic, and barrier-stabilizing gene networks, thereby transitioning vessels from growth to stabilization/maturation [43–45]. Our comparative analyses revealed temporal changes in PIEZO1/KLF2-related transcriptional features in hVOS ECs (Fig. 4G), consistent with a transition from a more angiogenic state toward a relatively stabilized endothelial state, suggesting dynamic regulation of endothelial responses within the 3D microenvironment.

To further define the cell-type-specific effects of 3D microenvironment, we compared day-7 and day-14 hVOS ECs and SMCs with their corresponding 2D cultures. hVOS ECs maintained endothelial markers such as PECAM1, CDH5 and showed increased vascular organization, mechanotransduction, and hypoxia-related gene expression, whereas proliferation markers such as MKI67, TOP2A were preferentially expressed in 2D ECs (Additional file 1: Figure S4A). Consistently, GO analysis showed enrichment of endothelial differentiation and angiogenesis, cytoskeletal and cell-matrix organization, and hypoxia-response programs, together with reduced cell-cycle and DNA-replication pathways (Additional file 1: Figure S4B, C). Similarly, hVOS SMCs exhibited increased expression of contractile markers, including ACTG2, TAGLN (Additional file 1: Figure S5A), accompanied by enrichment of muscle maturation, mechanosensitive cytoskeletal organization, ECM interaction, and hypoxia-response pathways. Proliferation-related genes and pathways were predominantly associated with 2D SMCs (Additional file 1: Figure S5B, C). Together, these analyses reveal cell-type-specific transcriptional signatures associated with vascular maturation, mechanotransduction, and hypoxic adaptation in hVOS ECs and SMCs.

In summary, hVOS exhibited transcriptional features associated with vascular organization, hypoxia responses, and mechanotransduction-related adaptation. These findings indicate that the 3D microenvironment is accompanied by the activation of maturation-associated vascular programs and suggest potential roles for multicellular interactions and environmental cues in this process.

### hVOS transplantation promoted functional recovery and tissue repair in a mouse model of hindlimb ischemia

**Fig. 5:**
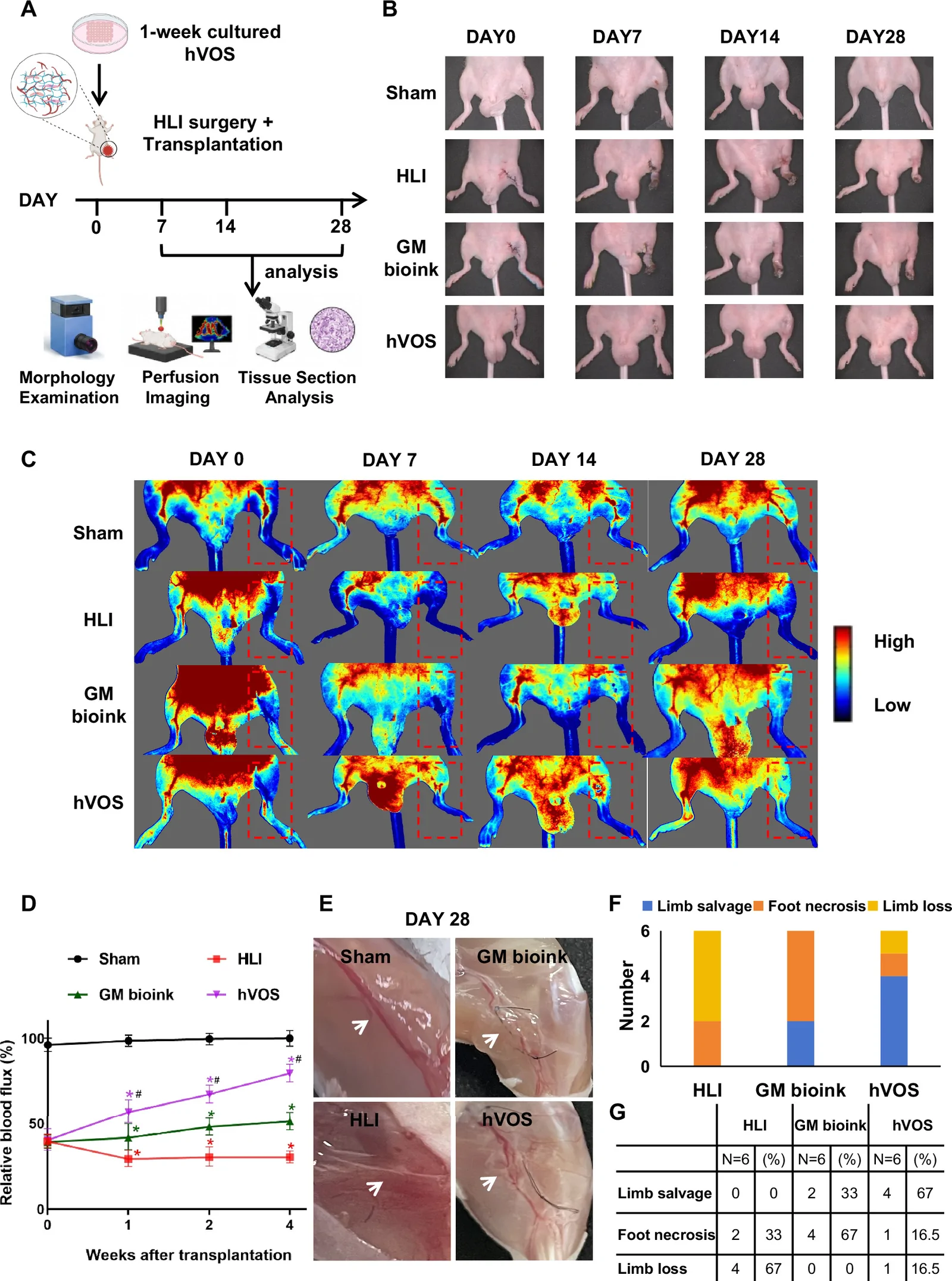
. hVOS transplantation promoted revascularization and limb salvage in hindlimb ischemia. **A** Schematic of hVOS production and transplantation in hindlimb ischemia (HLI) mouse models. **B** Representative images of the hindlimb from sham, HLI, GM bioink, and hVOS transplanted mice (*n* = 6 mice in each group). **C** Representative hind limb perfusion mapping images. **D** Quantification of blood flux/perfusion in hind limbs over time (right hind limb, 1, 2, and 4 weeks) in each group. Relative blood flux was calculated as the perfusion value of the treated limb (left) divided by that of the non-ischemic limb (right) (*P < 0.05 vs. sham. #*P* < 0.05 vs. HLI, two-way ANOVA with Tukey’s multiple-comparison test). **E** Photographs showing new blood vessel formation across ligation sites of the ischemic hindlimb 28 days after treatment. **F** Stacked bar graph showing the percentage distribution of the physiological status of treated ischemic limbs on day 28 post-implantation of HLI, GM bioink and hVOS. **G** The table listed the number and percentage of limb salvage outcomes of HLI, GM bioink, and hVOS group 28 days after transplantation

We next assessed the therapeutic potential of 3D bioprinted hVOS for repairing hindlimb ischemia. hVOS constructs were first cultured in vitro for 7 days to establish vascular networks before transplanting into a mouse model of hindlimb ischemia. The study included four groups: a sham operation group, HLI model group, GM bioink transplantation group, and hVOS transplantation group. Blood flow was measured at 1, 2, and 4 weeks after transplantation, followed by tissue collection, cryosectioning, H&E staining, and immunofluorescence analysis (Fig. 5A). Visual examination found substantial necrosis and tissue loss in the hindlimb of the HLI group, while both transplantation groups showed limb salvage, with the hVOS transplantation group exhibiting a markedly superior therapeutic effect compared to the GM bioink group (Fig. 5B, Additional file 1: Figures S6 and S7). Blood flow measurements and statistical analyses further confirmed that the hVOS transplantation group achieved significantly better therapeutic efficacy in blood flow recovery (Fig. 5C, D). By the fourth week post-transplantation, the damaged blood vessels in the hindlimbs of the hVOS transplantation group had recovered better, comparable to the control group (Fig. 5E). Furthermore, statistical analysis showed that 67% of mice in the hVOS transplantation group exhibited limb salvage, whereas no limb salvage was observed in the HLI group, and only 33% of mice in the GM bioink transplantation group achieved limb salvage (Fig. 5F, G).

**Fig. 6:**
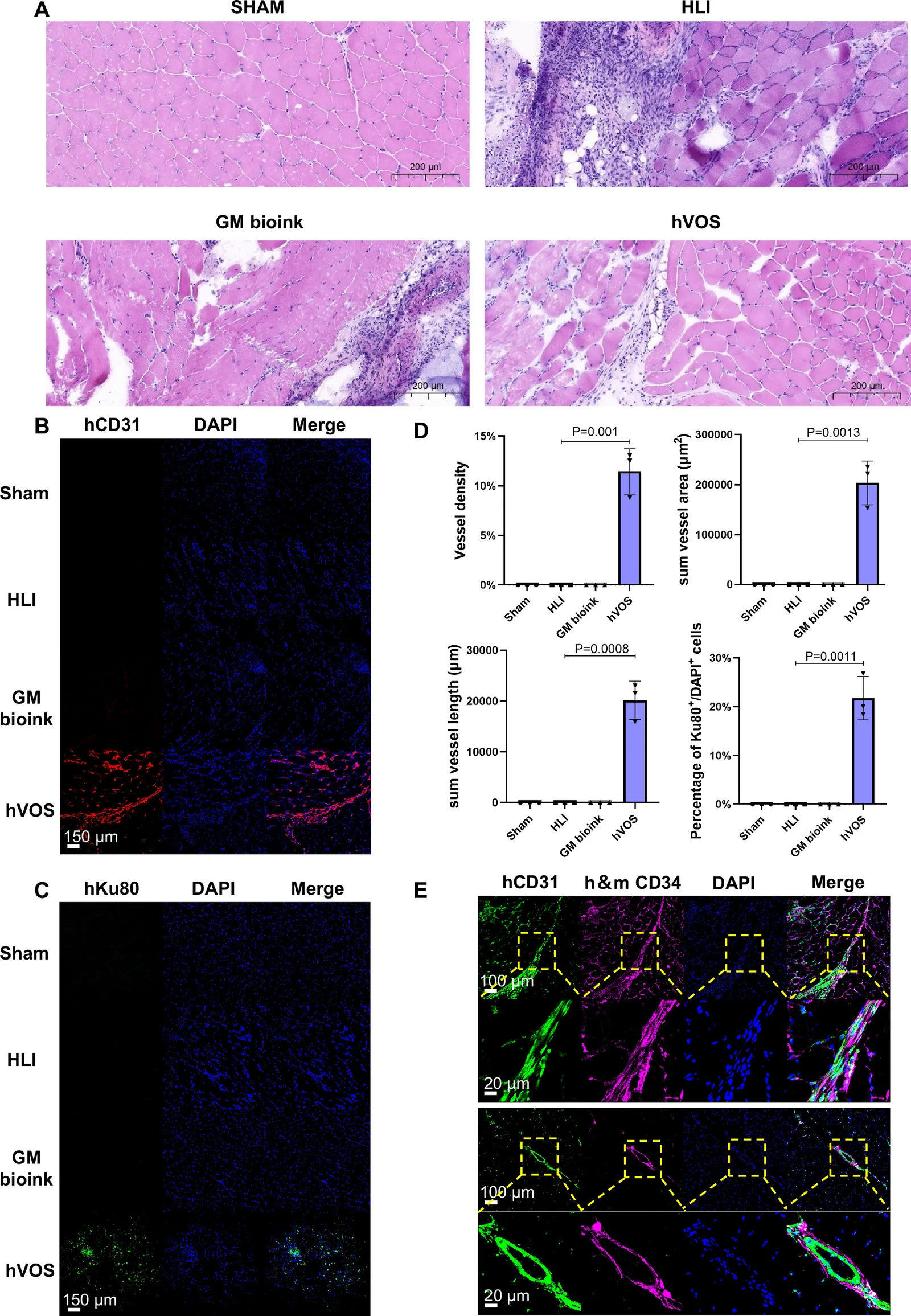
Transplanted hVOS formed human blood vessels in the ischemic hindlimb. **A** Representative H&E staining, **B** hCD31, and **C** hKu80 immunofluorescence of hindlimb tissue sections on day 7 after transplantation. **D** Quantitative analysis results of vessel density, sum vessel area, sum vessel length, percentage of Ku80^+^/DAPI^+^ cells (*n* = 3 biological replicates, two-tailed unpaired Student’s t-test). **E** Representative immunofluorescence images of hCD31 (green) and CD34 (pink) on day 7 after transplantation. Scale bar is as indicated

H&E staining of the frozen tissue sections from day 7 showed that, compared to the control group, the hVOS transplantation group showed reduced inflammation and immune cell infiltration. Over time, immune infiltration in the hVOS transplantation group further declined, and tissue morphology gradually returned to normal (Fig. 6A, Additional file 1: Figure S8A). To determine whether human cells within the hVOS contributed to vascular formation in mice, we used human-specific CD31 and Ku80 antibodies to perform immunofluorescence staining on frozen tissue sections from each group on day 7 post-transplantation. The results showed that, compared to the control group, the hVOS transplantation group had widespread expression of hCD31 and hKu80 (Fig. 6B, C). Statistical analysis revealed that the hVOS transplantation group had significantly higher vascular density, vascular area, vessel length, and proportion of hKu80-positive cells (Fig. 6D). Based on these, we performed double immunostaining with a CD34 antibody, which stained mouse and human vascular cells, and hCD31 antibody. The result suggested that ECs in transplanted hVOS contribute to vascular remodeling after transplantation (Fig. 6E). Although the proportion of hKu80-positive cells and hCD31 gradually decreased over time, human-derived cells were still present in the hindlimb tissues of the hVOS transplantation group up to 28 days post-transplantation (Additional file 1: Figure S8B-E).

**Fig. 7:**
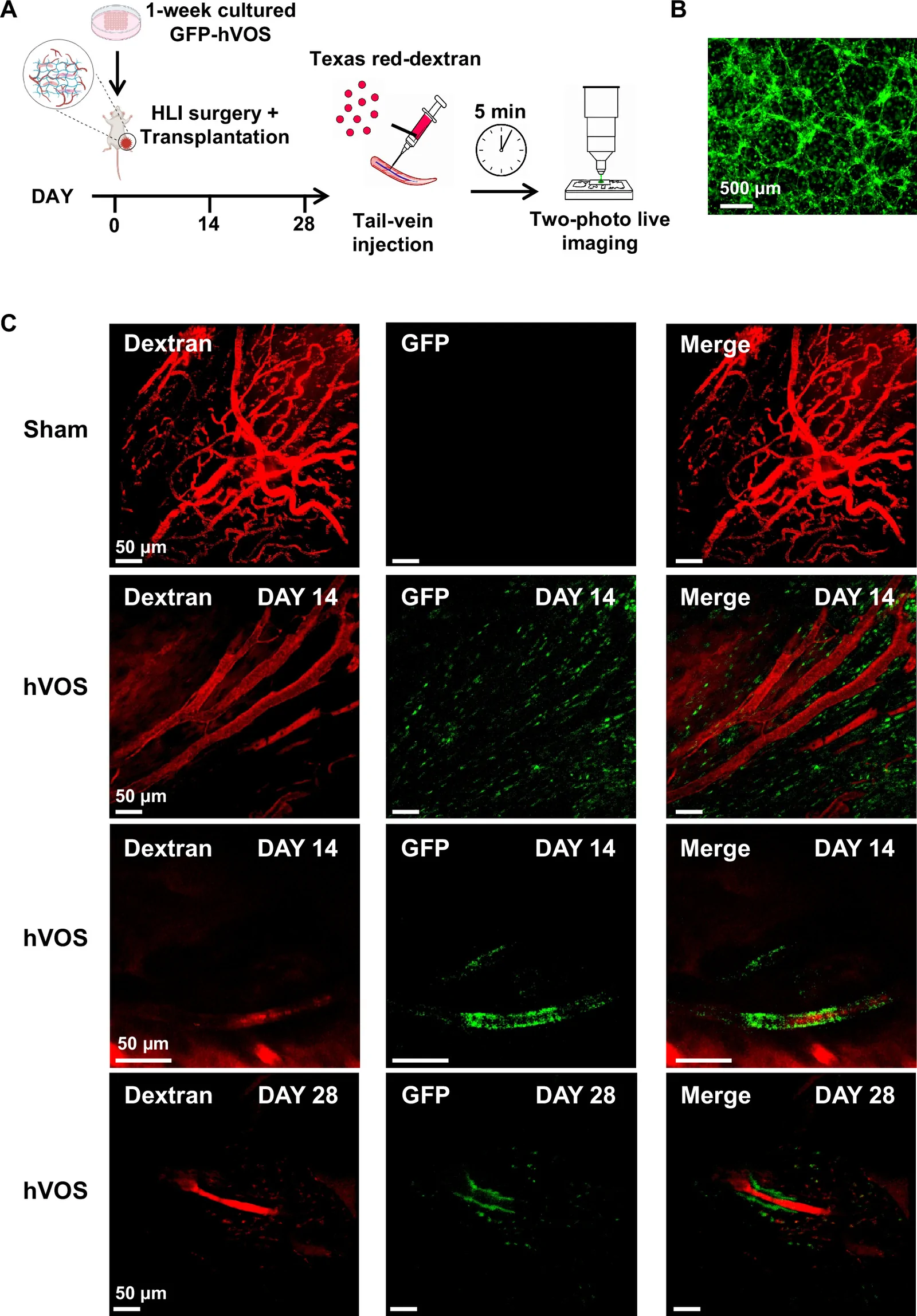
Intravital two-photon imaging demonstrates functional perfusion of transplanted hVOS-derived vascular structures by the host circulation. **A** Schematic illustration of GFP-labeled hVOS bioprinting, transplantation, intravenous Texas Red–dextran administration, and intravital two-photon imaging. **B** Representative fluorescence image of a bioprinted GFP-labeled hVOS prior to transplantation. **C** Representative intravital two-photon images showing the Texas Red–dextran channel (host circulation), GFP channel (transplanted hVOS-derived vascular structures), and merged images at 14 and 28 days after GFP-hVOS transplantation

To further determine whether hVOS-derived vascular structures established functional connections with the host circulation, we performed intravital two-photon imaging following transplantation of GFP-labeled hVOS generated from ECs and SMCs differentiated from the GFP-H1 cell line (Fig. 7A, B). Following intravenous injection of fluorescent dextran, GFP-positive hVOS-derived vascular structures showed circulation-associated dextran signals at days 14 and 28 after transplantation (Fig. 7C, Additional file 2: Supplementary Video 1, Additional file 3: Supplementary Video 2), The presence of circulating dextran within GFP-positive vascular structures indicates that hVOS-derived vessels were perfused by the host circulation, providing further evidence of their functional vascular integration after transplantation.

### ScRNA-seq revealed adaptive remodeling of transplanted human cells within the ischemic microenvironment

**Fig. 8:**
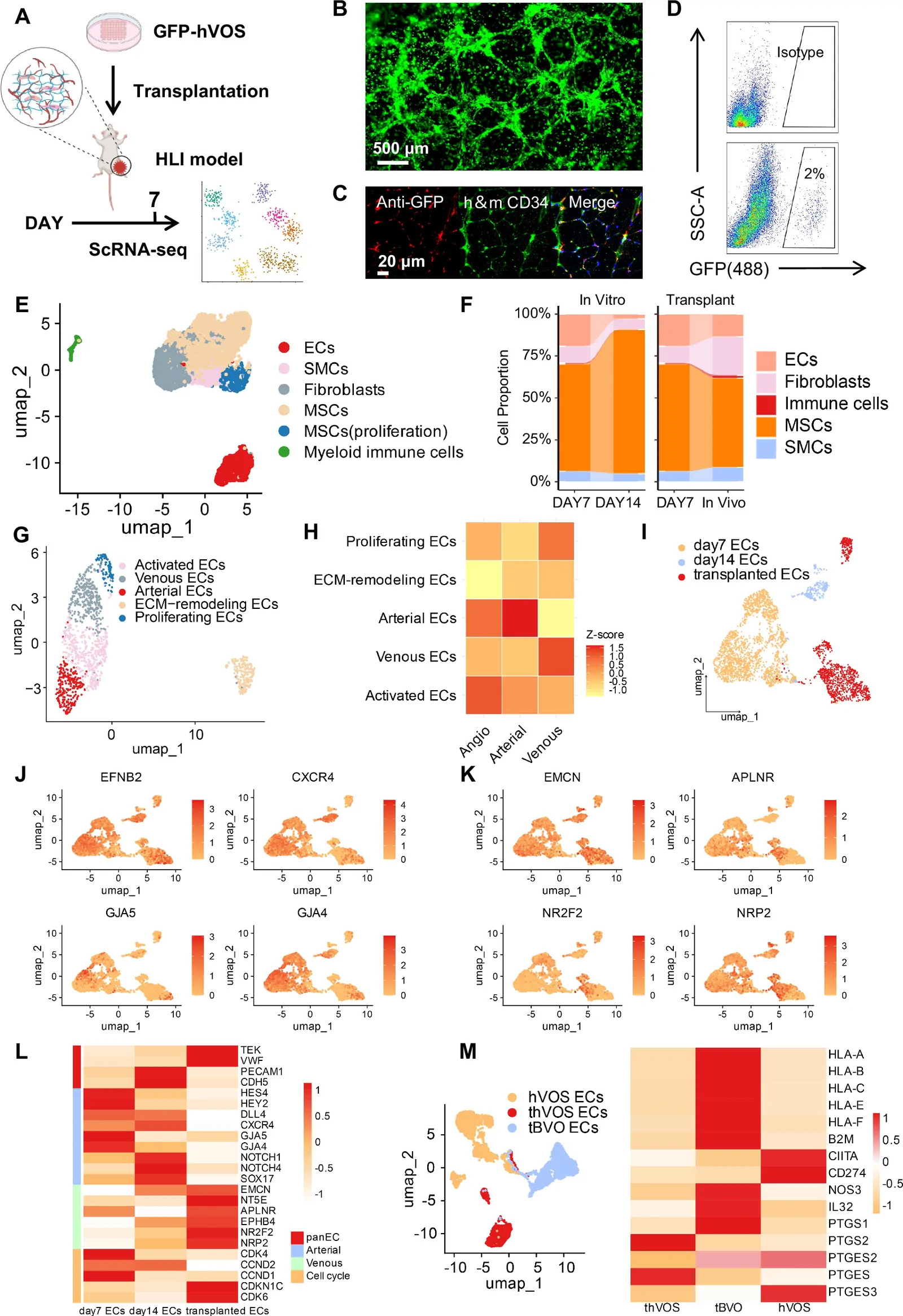
ScRNA-seq analysis of transplanted hVOS. **A** Schematic of hVOS transplantation and cell harvest. **B** Representative fluorescence image of GFP-labeled hVOS. **C** Co-immunostaining of GFP (red) and CD34 (green). **D** Flow cytometry quantification of GFP positive cells in harvested tissue. **E** UMAP visualization of transplanted hVOS cells at day 7. **F** Bar graph showing changes in the cell proportion. **G** UMAP visualization of EC subtypes at day 7. **H** Heatmap of angiogenesis, arterial, and venous signature scores across EC subtypes. **I** UMAP visualization of the ECs populations from day 7, day 14, and transplanted hVOS. **J-K** Feature plot showing expression of arterial- and venous-specific marker genes. **L** Heatmap of EC-related gene expression. **M** Left: UMAP comparing EC populations from in vitro hVOS, transplanted hVOS (thVOS), and transplanted BVO (tBVO). Right: Heatmap of immune-related gene expression

To investigate the impact of the in vivo HLI microenvironment on hVOS, we transplanted GFP-labeled hVOS, into a mouse model of HLI. One week after transplantation, GFP-positive cells were sorted and subjected to scRNA-seq analysis (Fig. 8A). GFP-hVOS exhibited strong GFP fluorescence, and by day 7 in vitro, vascular networks had already begun to form (Fig. 8B). At 7 days post-transplantation, immunofluorescence analysis revealed successful integration between hVOS-derived cells and host mouse cells (Fig. 8C). GFP-positive cells were subsequently sorted by flow cytometry for single-cell RNA sequencing (Fig. 8D).

Analysis of the sequencing data revealed six distinct cell populations: ECs (13.4%), SMCs (8.53%), fibroblasts (23.1%), MSCs (47.0%), proliferating MSCs (6.43%), and myeloid immune cells (1.58%) (Fig. 8E, Additional file 1: Figure S9A, B). Comparative analysis of cell type proportions showed that the proportion of ECs was significantly higher in the in vivo group than in the in vitro day 14 group (13.4% versus 3.3%) (Fig. 8F).

Within the EC population, scRNA-seq revealed substantial transcriptional heterogeneity and identified five distinct endothelial states among the engrafted human cells (Figs. 8G and 8H). One subtype, termed “Activated ECs” was characterized by high expression of ESM1 and NEDD4L, two markers associated with activated endothelial states [46, 47]. GO enrichment analysis revealed significant enrichment of pathways related to immune response-activating signaling and positive regulation of cell adhesion (Additional file 1: Figure S9C, D), consistent with an endothelial state responsive to the inflammatory and reparative cues of the ischemic microenvironment. Notably, ECs recovered from transplanted hVOS exhibited a degree of endothelial-state remodeling comparable to that previously reported for BVOs transplanted under the kidney capsule [48] (Additional file 1: Figure S9E, F), indicating that substantial endothelial adaptation is a common feature of vascular organoids following engraftment.

We next examined whether transplantation altered endothelial functional state. Comparison of ECs from hVOS cultured for 7 or 14 days in vitro with ECs recovered after transplantation revealed increased expression of venous-associated genes in the transplanted ECs (Fig. 8I–L) [16, 49, 50]. To systematically define these changes, we performed UCell-based scoring [51] of arterial, venous, capillary, angiogenic, inflammatory, and cell-cycle transcriptional programs using the signature genes listed in Supplementary Table 1 (Additional file 4: Supplementary Table 1) [28, 52–56]. Transplanted ECs exhibited a reduced arterial-associated signature together with increased venous-associated and inflammatory signatures, whereas capillary, angiogenic, and cell-cycle programs remained largely unchanged (Additional file 1: Figure S10A, B). Consistent with the signature-level analysis, arterial-associated markers CXCR4, GJA4, and EFNB2 were broadly expressed in hVOS ECs before transplantation but became restricted to a subset of ECs after transplantation. In contrast, venous-associated markers NR2F2, APLNR, and NT5E, which were initially expressed in a smaller EC population, became enriched after transplantation, particularly among ECs with lower expression of arterial-associated markers (Additional file 1: Figure S10C). These findings indicate a shift in endothelial state toward a venous-associated phenotype following exposure to the ischemic microenvironment.

To further validate this transcriptional remodeling at the protein level, we performed flow cytometric analysis using human CD31 (hCD31) together with CXCR4 and NT5E as representative arterial- and venous-associated surface markers. In day 7 hVOS before transplantation, 14.3% of cells were hCD31⁺CXCR4⁺, whereas only 1.63% were hCD31⁺CXCR4⁻NT5E⁺, indicating that the majority of ECs exhibited an arterial-associated phenotype. In contrast, analysis of cells isolated from the transplanted region at day 7 after implantation showed a marked reduction in hCD31⁺CXCR4⁺ cells to 0.024%, whereas hCD31⁺CXCR4⁻NT5E⁺ cells accounted for 0.035%. Accordingly, the venous-to-arterial EC ratio increased after transplantation (Additional file 1: Figure S10D), providing independent flow cytometric evidence for the venous-biased endothelial remodeling identified by scRNA-seq.

We further characterized inflammation- and stress-associated transcriptional responses in transplanted ECs using the signature genes listed in Supplementary Table 2 (Additional file 5: Supplementary Table 2). NF-κB-associated [57] and stress-response[42, 58] signatures were increased following transplantation, whereas prostaglandin-related genes [59] remained minimally expressed and interferon [60] and cytokine-response [61] signatures showed limited changes (Additional file 1: Figure S11A–D). Thus, transplanted ECs adopted a transcriptional state characterized predominantly by NF-κB- and stress-associated endothelial state after transplantation rather than broad prostaglandin- or interferon-associated activation.

We also examined the expression levels of HLA family genes related to immune rejection in ECs. Notably, compared with in vitro-cultured hVOS and kidney capsule-transplanted Matrigel-based BVO [48], ECs recovered from hVOS transplanted into the HLI model exhibited reduced expression of selected HLA- and antigen presentation-related genes (Fig. 8M), suggesting altered immune-related gene expression patterns after transplantation.

We also examined transcriptional changes in non-endothelial populations. Comparative analysis of SMCs and fibroblasts from transplanted hVOS versus in vitro-cultured hVOS revealed significant enrichment of pathways related to wound healing and extracellular matrix remodeling in the transplanted cells (Additional file 1: Additional file 1: Figure S9G-J). Similar increases in NF-κB-associated and stress-response signatures were observed in transplanted SMCs and fibroblasts, while prostaglandin- and interferon-related signatures remained low (Additional file 1: Figure S11E, F). These findings indicate that adaptive transcriptional remodeling in response to the ischemic microenvironment extends beyond ECs to stromal populations, suggesting coordinated contributions of multiple hVOS-derived cell types to tissue repair following transplantation.

## Discussion

In this study, we successfully engineered human vascular organoid sheets (hVOS) by 3D bioprinting hPSC-ECs and hPSC-SMCs using GelMA-Matrigel bioinks. The hVOS recapitulated key features of stable and complex vascular networks and exhibited functional and molecular characteristics consistent with those of native blood vessels. When transplanted into a murine hindlimb ischemia model, the hVOS successfully formed blood vessels in vivo with host mouse cells, significantly promoting functional recovery and tissue regeneration in the ischemic hindlimb.

Our results support an important role for SMCs in the organization and long-term stability of hVOS. Previous studies have demonstrated that mural cells promote vascular stabilization and maturation [62–64], whereas earlier BVO models did not incorporate SMCs [7]. In our system, ECs and SMCs were incorporated at a predefined ratio during construct fabrication. A preliminary comparison of EC:SMC ratios (3:1, 2:1, and 1:1) identified the 2:1 ratio producing the most continuous and interconnected vascular networks, and was therefore selected for subsequent hVOS fabrication. Nevertheless, optimization was limited to the current bioink formulation and construct design. Because the optimal cellular composition is likely influenced by factors such as bioink properties, cell types,total cell density, and construct geometry, future studies will require systematic optimization of these parameters for different vascular tissue engineering applications.

Moreover, the differentiation stage of ECs and SMCs significantly impacts the successful assembly and functionality of the hVOS. While many studies have employed adult HUVECs as seed cells for bioprinting, these cells have been reported to form vascular structures that lack long-term stability[65]. In contrast, early vascular cells derived from hPSCs offer significant advantages [66, 67]. These cells are reported to exhibit superior proliferative capacity and differentiation potential, providing greater plasticity than adult cells. This enhanced plasticity facilitates the formation of more physiologically relevant vascular structures, underscoring the potential of hPSC-derived early vascular cells to generate stable, mature vascular networks.

Our scRNA-seq analysis revealed that hVOS contained not only ECs and SMCs but also fibroblasts, MSCs, and a small macrophage population. Fibroblasts and MSCs are known to support vascular maturation and remodeling [68, 69]. while macrophages can actively regulate angiogenesis, vascular anastomosis [70]. Pro-angiogenic macrophages may promote endothelial sprouting and network formation through paracrine signaling and extracellular-matrix remodeling, including VEGFA secretion, MMP-dependent matrix remodeling, and TGF-β/SPP1-related pathways [71–73]. Therefore, the macrophage-like population emerging within hVOS may contribute to vascular network assembly by supporting endothelial migration, matrix remodeling, and EC–mural cell interactions. However, their specific functional contribution remains to be determined. Future studies could explore controlled incorporation or enrichment of macrophages during hVOS fabrication to further investigate their role in vascular development and remodeling.

To date, few studies have characterized the transcriptional remodeling of engineered human vascular cells after transplantation. In our study, the pronounced remodeling of graft-derived EC states indicates that the ischemic microenvironment exerts a strong influence on endothelial adaptation after transplantation. Transplanted ECs displayed a venous-biased adaptive phenotype accompanied by endothelial activation, indicating that vascular specification within hVOS remains plastic and responsive to the local tissue environment. The concurrent NF-κB- and stress-associated changes observed in ECs, SMCs, and fibroblasts further suggest a coordinated adaptive response to ischemic injury, together with stromal programs involved in wound healing and extracellular-matrix remodeling. These findings highlight the importance of the host microenvironment in directing the post-transplantation remodeling of engineered vascular tissues.

Reduced expression of selected HLA- and antigen-presentation-related genes was also observed in transplanted hVOS-derived ECs. However, because transplantation was performed in immunodeficient mice, these transcriptional changes cannot be interpreted as evidence of reduced immunogenicity or immune tolerance and may instead reflect differences in cellular state or local microenvironment. Further validation in appropriate immune models will be required to define the immunological properties of hVOS.

The hVOS presents several distinct advantages. First, unlike Matrigel-based hPSC-derived BVOs, which typically form capillary-like networks lacking mural cell coverage [74]. hVOS incorporates hPSC-derived ECs and SMCs at a predefined ratio from the beginning of construct fabrication. This design provides greater control over cellular composition and supports the formation of endothelial networks associated with SMC coverage.

Second, hVOS differs from conventional EC/SMC co-transplantation approaches. In many co-transplantation studies, ECs and SMCs are delivered as cell suspensions or matrix-embedded mixtures, and vascular assembly occurs mainly after implantation. In contrast, hVOS is transplanted as a structured organoid sheet containing a preformed vascular network, thereby preserving a defined cellular composition and spatial organization at the time of implantation. The sheet format also facilitates retention of the construct at the ischemic site and permits localized delivery of the engineered vascular tissue.

Third, hVOS differs from previously reported 3D-bioprinted vascular patches. Recent EC/SMC-containing bioprinted patches have shown therapeutic benefits in hindlimb ischemia, but some of these constructs used primary umbilical cord-derived vascular cells [75] rather than hPSC-derived vascular cells and were developed primarily as cell-laden patches rather than hPSC-derived vascular organoid sheets with a preassembled multicellular network. Moreover, post-transplantation remodeling of graft-derived cells has rarely been examined at single-cell resolution in these systems. In the present study, scRNA-seq of recovered human cells enabled assessment of how graft-derived cells states changed within the ischemic microenvironment.

Thus, the novelty of hVOS lies in the integration of several complementary features: the use of hPSC-derived ECs and SMCs at a controlled ratio, fabrication of a reproducible organoid-sheet architecture by 3D bioprinting, implantation of a construct containing a preformed vascular network, functional evaluation in hindlimb ischemia, and single-cell characterization of graft-derived human cells after transplantation. Rather than representing an entirely unprecedented vascular-cell or bioprinting strategy, hVOS combines engineered construct control, therapeutic evaluation, and post-transplantation cellular-state analysis within one experimental platform. This integrated framework provides a useful system for studying how engineered human vascular tissues respond to ischemic environments.

Several limitations remain. Although intravital imaging provided evidence that hVOS-derived vascular structures participate in host-associated perfusion, the detailed cellular organization of the host–graft vascular interface remains to be resolved. In addition, the hypoxia/HIF- and PIEZO1/KLF2-related programs identified in this study were inferred from transcriptomic and imaging analyses and require functional validation by targeted perturbation. Comprehensive reconstruction of vascular architecture across the entire hVOS [76]also remains challenging [77] because of the relatively large construct size and soft hydrogel composition. Moreover, clinically relevant factors such as aging, diabetes, and an intact immune system were not modeled. Future studies should therefore focus on improving construct scalability and perfusion, [22, 78] validating key molecular mechanisms, and evaluating long-term efficacy, safety, and immune compatibility in more physiologically [28, 79]relevant models.

Beyond addressing these limitations, the hVOS platform may provide a versatile system for both regenerative and translational applications. Its controllable cellular composition and geometry could facilitate the development of patient-specific vascular constructs for ischemic tissue repair, while its defined multicellular architecture may also support vascular disease modeling and drug screening. In addition, the ability to recover and analyze graft-derived human cells after transplantation provides an opportunity to investigate how engineered vascular tissues adapt to distinct in vivo [48, 80] microenvironments.

## Conclusions

In summary, we developed a reliable and architecturally adjustable 3D bioprinting method to construct hVOS with well-developed vascular structures and enhanced maturation. Using single-cell transcriptomic analysis, we gained insights into the biological characteristics and development of these structures. Furthermore, we demonstrated the therapeutic potential of these 3D bioprinted hVOS for treating hindlimb ischemia, laying a promising foundation for future advancements in transplantation-based therapies.

## Supplementary information

Supplementary Information

Supplementary Additional file 2

Supplementary Additional file 3

Supplementary Additional file 4

Supplementary Additional file 5

Supplementary Information

## Data Availability

The raw sequencing data generated in this study have been deposited in the Genome Sequence Archive (GSA) for Human [76] at the National Genomics Data Center (NGDC), China National Center for Bioinformation/Beijing Institute of Genomics, Chinese Academy of Sciences [77], under accession number HRA013820. These data are publicly accessible at https://ngdc.cncb.ac.cn/gsa-human.The 2D-cultured hPSC-derived EC and SMC scRNA-seq data used in this study were obtained from the Gene Expression Omnibus (GEO) under accession number GSE166462 (https://identifiers.org/ncbi/geo:GSE166462) [22, 78]. The human vascular cell scRNA-seq dataset was downloaded from https://www.vascularcellatlas.org/#data [28, 79]. The BVO scRNA-seq dataset deposited in Zenodo under https://doi.org/10.5281/zenodo.14002044 [48, 80].

## Abbreviations

3D: Three-dimensional
2D: Two-dimensional
AATS: Albumin, ascorbic acid, transferrin, and sodium selenite
ANOVA: Analysis of variance
APC-Cy7: Allophycocyanin–cyanine 7
Bfgf: Basic fibroblast growth factor
BMP4: Bone morphogenetic protein 4
BVO: Blood vessel organoid
CCK-8: Cell Counting Kit-8
Cryo-SEM: Cryogenic scanning electron microscopy
DAPI: 4′,6-Diamidino-2-phenylindole
DNA: Deoxyribonucleic acid
EC: Endothelial cell
ECM: Extracellular matrix
EDTA: Ethylenediaminetetraacetic acid
FACS: Fluorescence-activated cell sorting
FBS: Fetal bovine serum
FCS: Fetal calf serum
FITC: Fluorescein isothiocyanate
GelMA: Gelatin methacryloyl
G-M: GelMA–Matrigel
GFP: Green fluorescent protein
GO: Gene Ontology
H&E: Hematoxylin and eosin
HIF: Hypoxia-inducible factor
HLA: Human leukocyte antigen
HLI: Hindlimb ischemia
hPSC: Human pluripotent stem cell
hiPSC: Human induced pluripotent stem cell
HUVEC: Human umbilical vein endothelial cell
hVOS: Human vascular organoid sheet
IACUC: Institutional Animal Care and Use Committee
IgG: Immunoglobulin G
LAP: Lithium phenyl-2,4,6-trimethylbenzoylphosphinate
MSC: Mesenchymal stromal cell
NF-κB: Nuclear factor kappa B
OCT: Optimal cutting temperature
PBS: Phosphate-buffered saline
PDGF: Platelet-derived growth factor
PDGF-BB: Platelet-derived growth factor-BB
PE: Phycoerythrin
RNA: Ribonucleic acid
ROCK: Rho-associated coiled-coil-containing protein kinase
RPMI: Roswell Park Memorial Institute
scRNA-seq: Single-cell RNA sequencing
SD: Standard deviation
SEM: Standard error of the mean
SMC: Smooth muscle cell
TC: Transition-state cell
TGF-β: Transforming growth factor beta
UMAP: Uniform manifold approximation and projection
UMI: Unique molecular identifier
VE-cadherin: Vascular endothelial cadherin
VEGF: Vascular endothelial growth factor
VEGF-A: Vascular endothelial growth factor A
WM: Wash medium.
