# Supplementary material for: 3D bioprinted human vascular organoid sheets promote functional ischemic repair and exhibit adaptive in vivo remodeling
Source: BMC Med. 2026 Sep 25;24:524. doi: 10.1186/s12916-026-05256-2 (PMC13621672; doi:10.1186/s12916-026-05256-2)

Figure S1. Characterization of the GelMA+Matrigel Hydrogel.

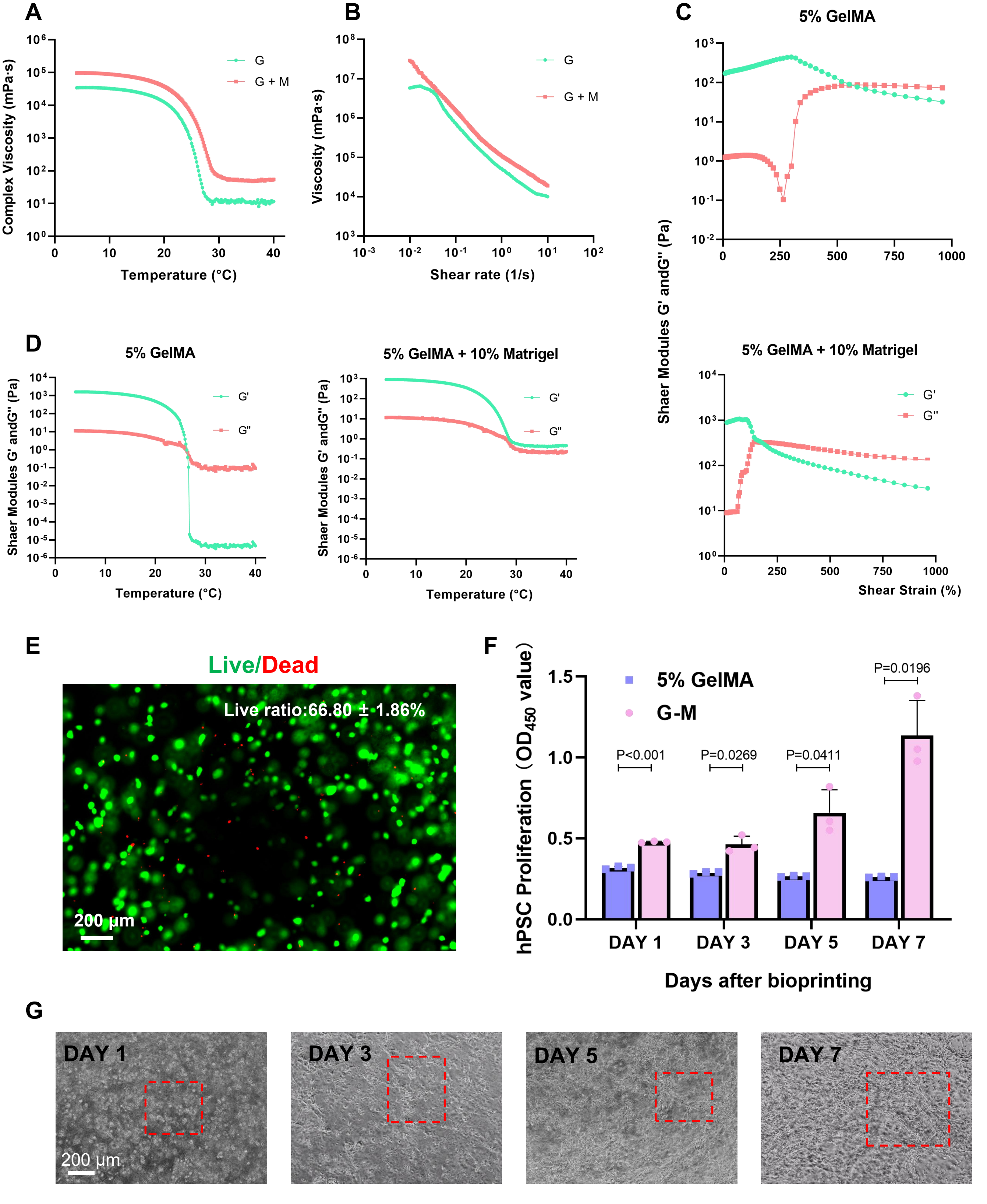

Figure S2. Optimization of cell ratio for hVOS fabrication.

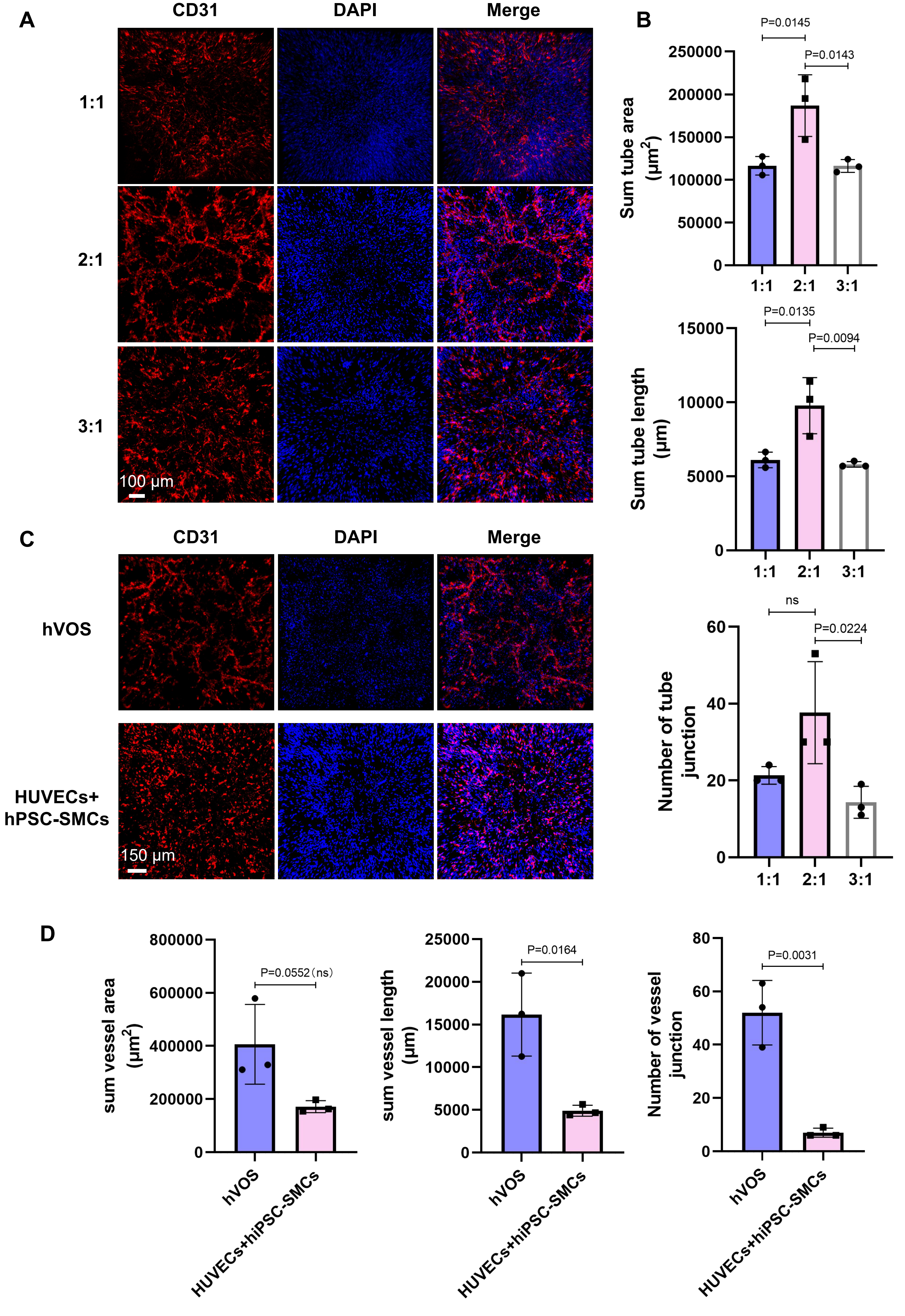

Figure S3. GO and comparative analysis of cell populations in bioprinted hVOS.

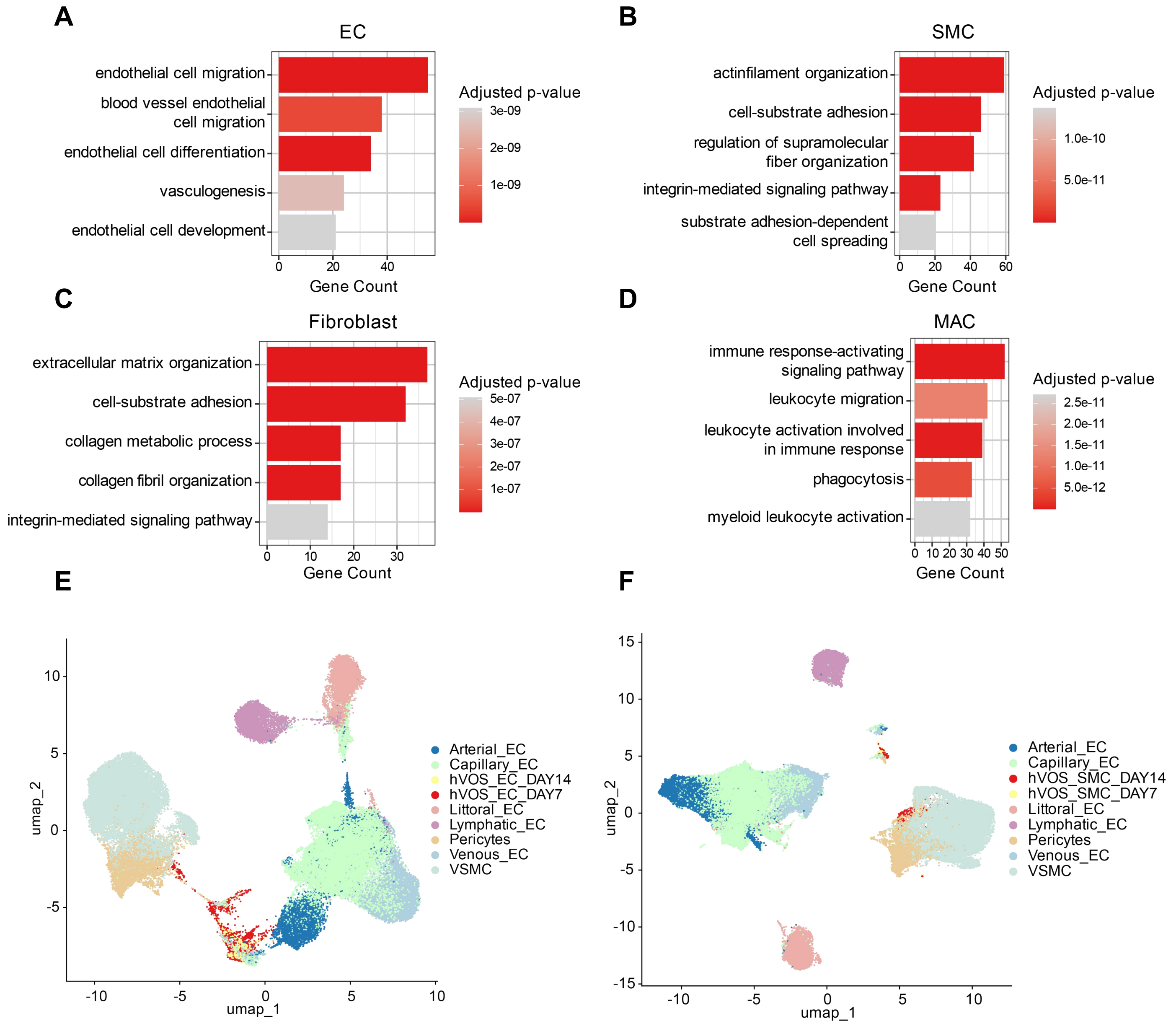

Figure S4. Transcriptomic comparison of day-7 and day-14 hVOS ECs with 2D-cultured ECs.

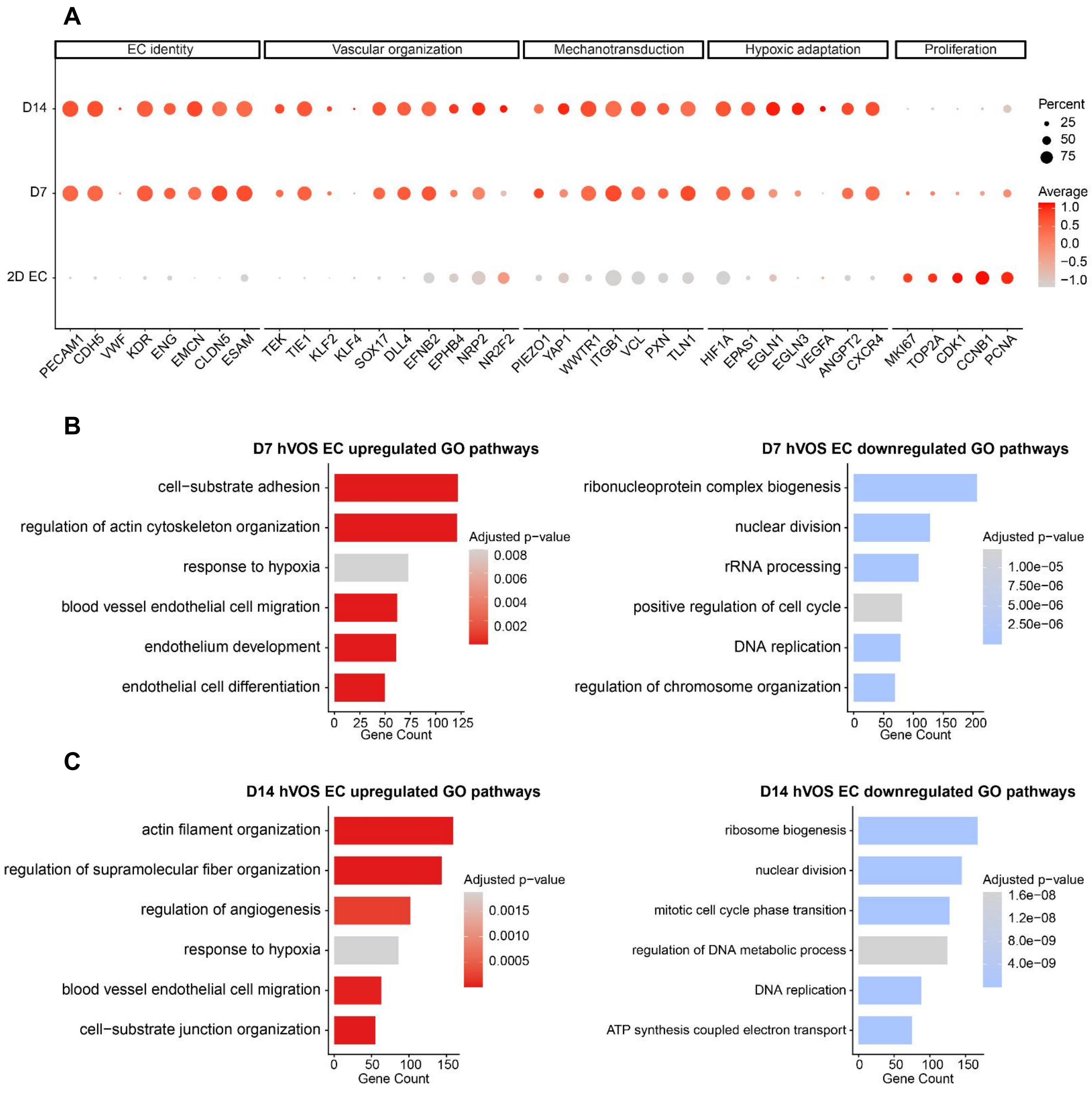

Figure S5. Transcriptomic comparison of day-7 and day-14 hVOS SMCs with 2D-cultured SMCs.

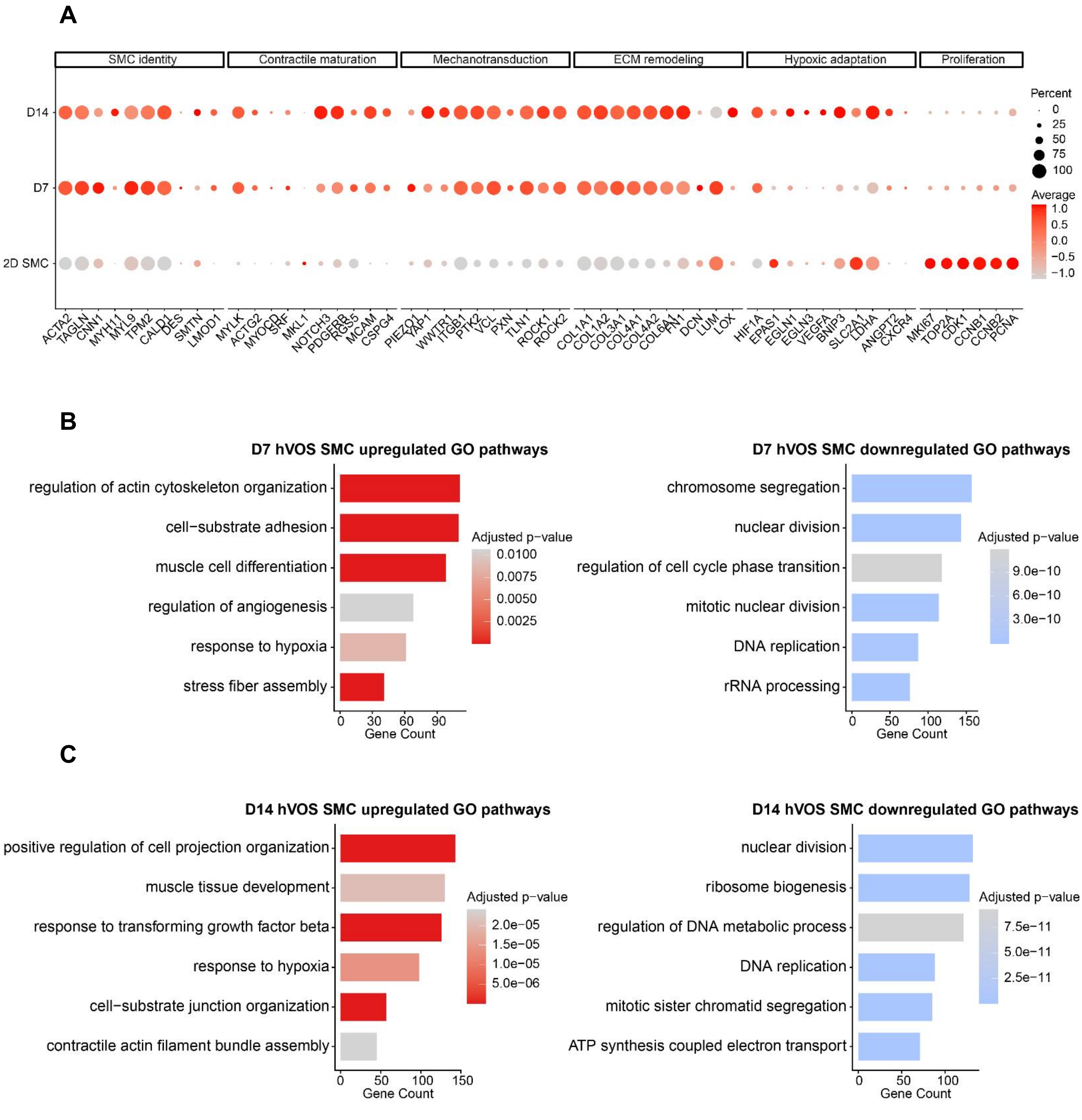

Figure S6. Images of the hindlimb from sham and HLI mice.

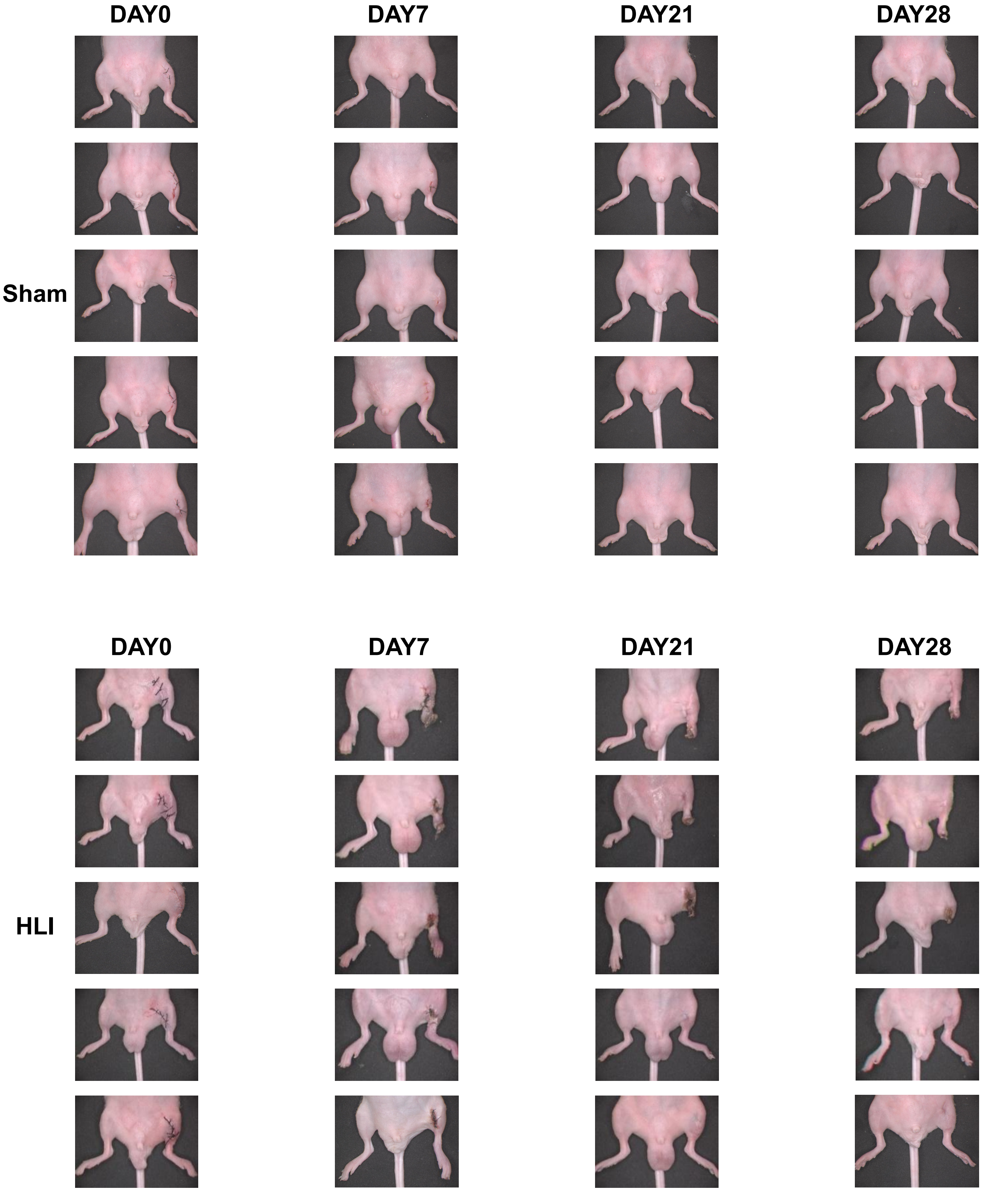

Figure S7. Images of the GM bioink and 3D bioprinted hVOS transplanted mice.

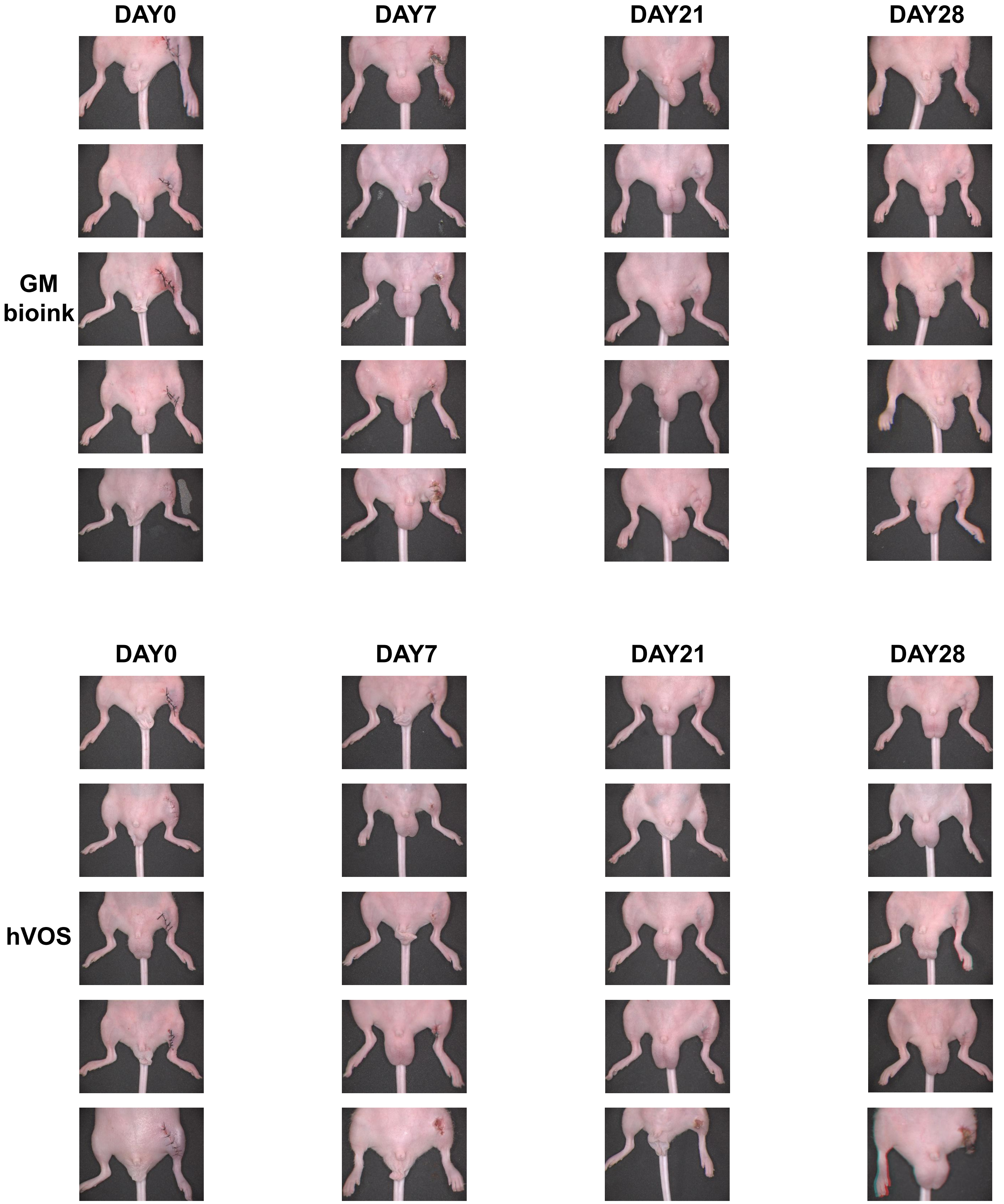

Figures S8. Transplanted hVOS reconstructs tissue architecture and forms functional human vasculature in ischemic hindlimb.

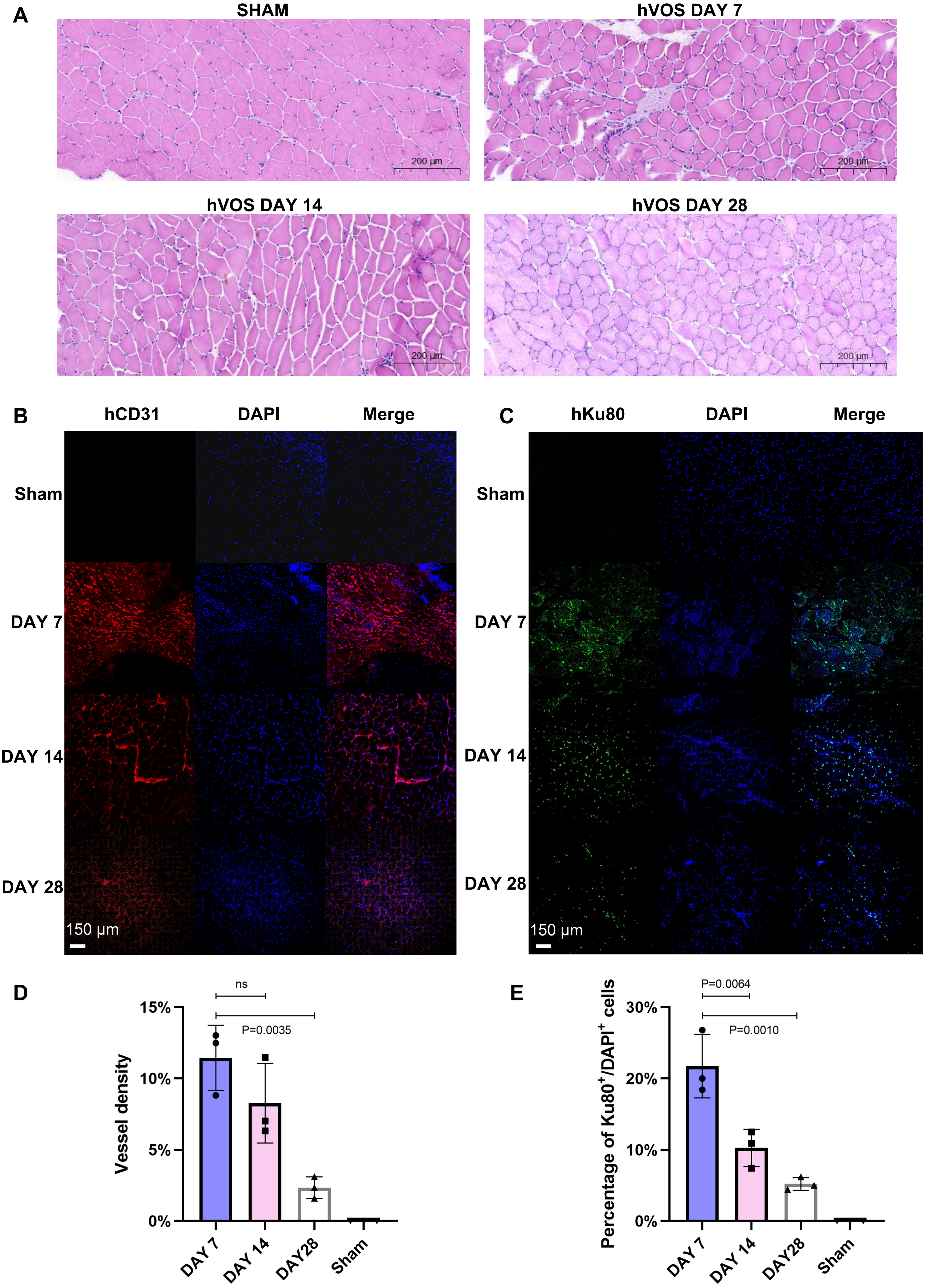

Figure S9. ScRNA-seq analysis of different cell types in hVOS following transplantation.

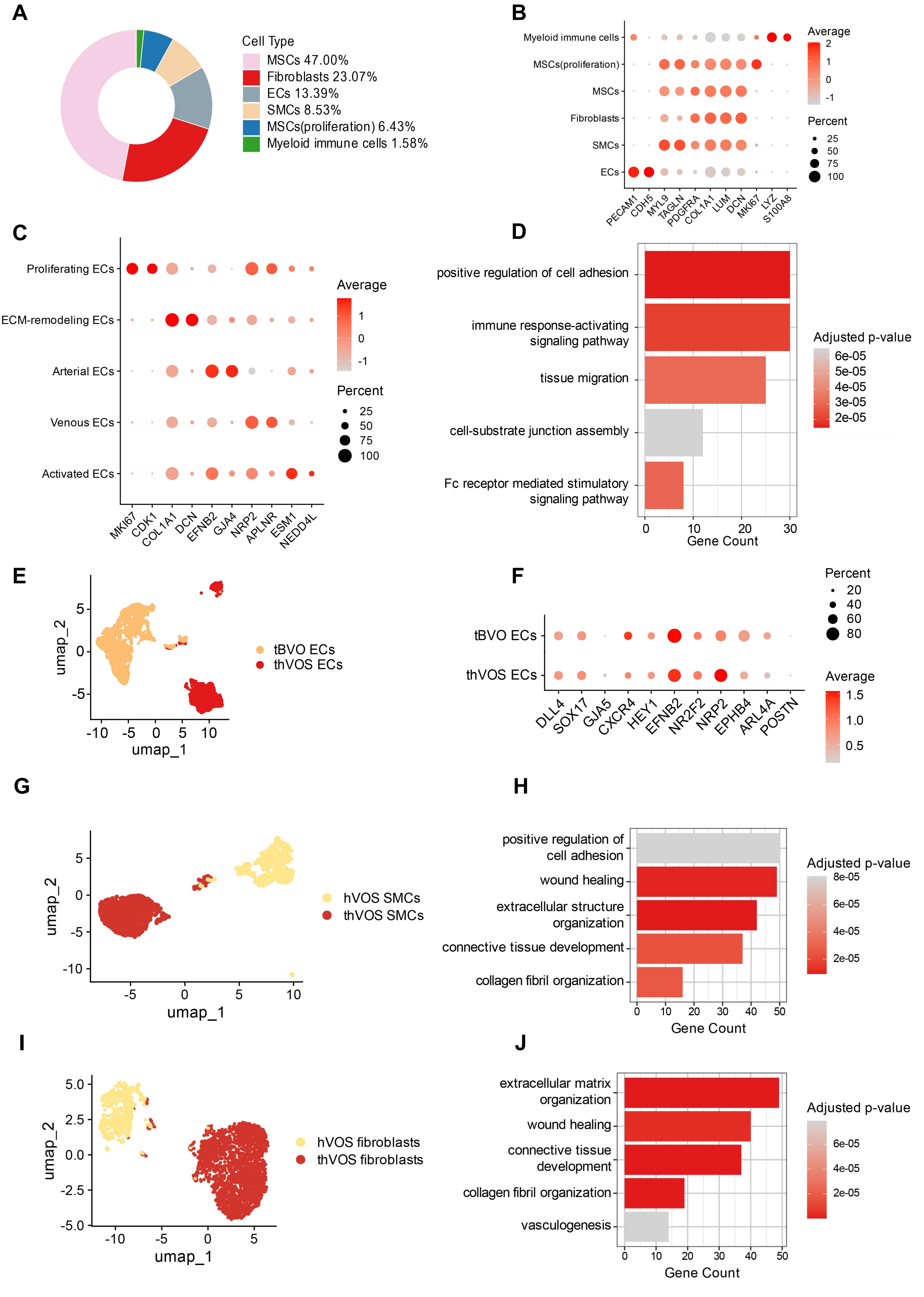

Figure S10. Transplantation induces a venous-biased endothelial remodeling state.

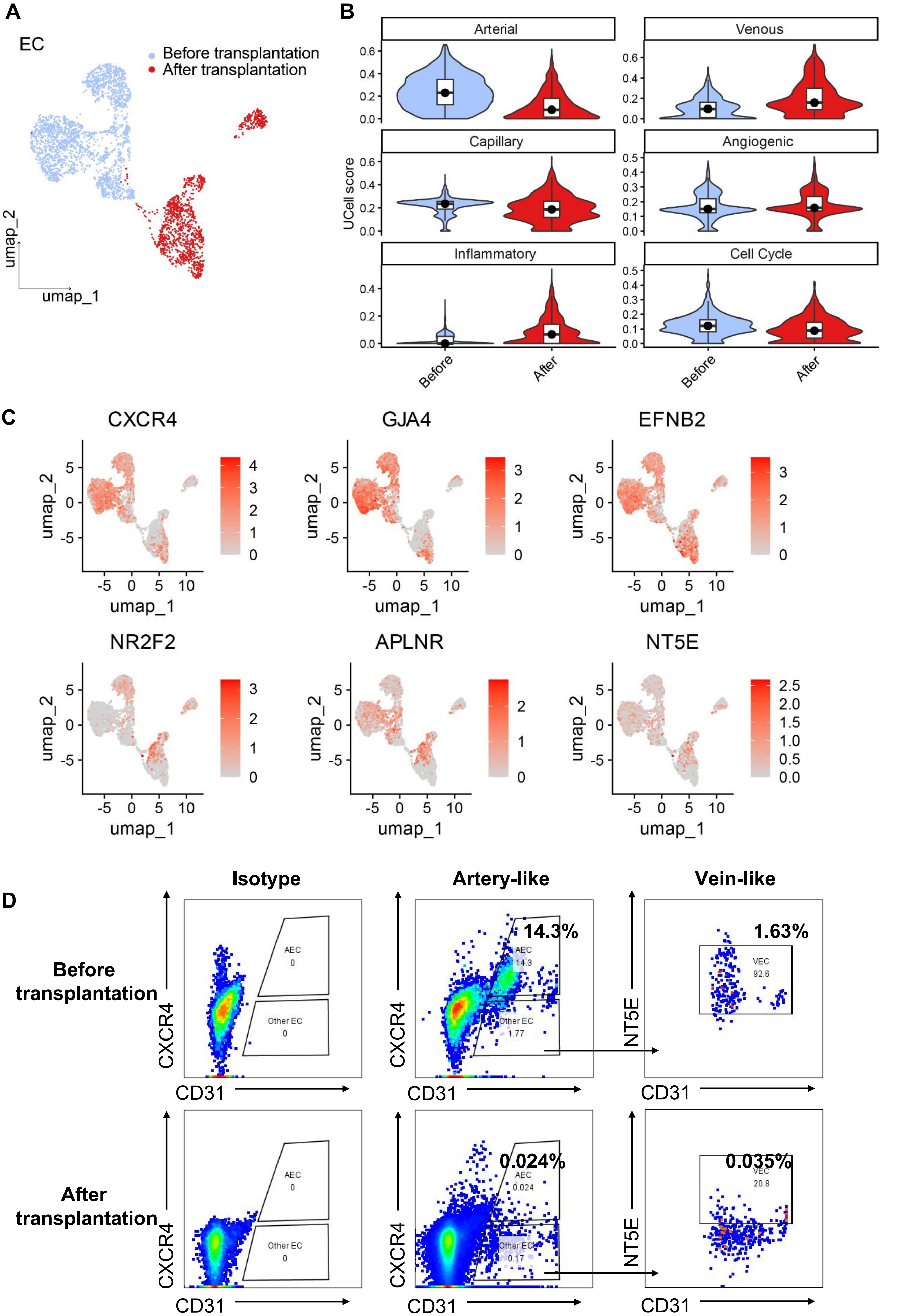

Figure S11. Transcriptional profiling reveals inflammatory and stress-response signatures in transplanted endothelial and stromal populations.

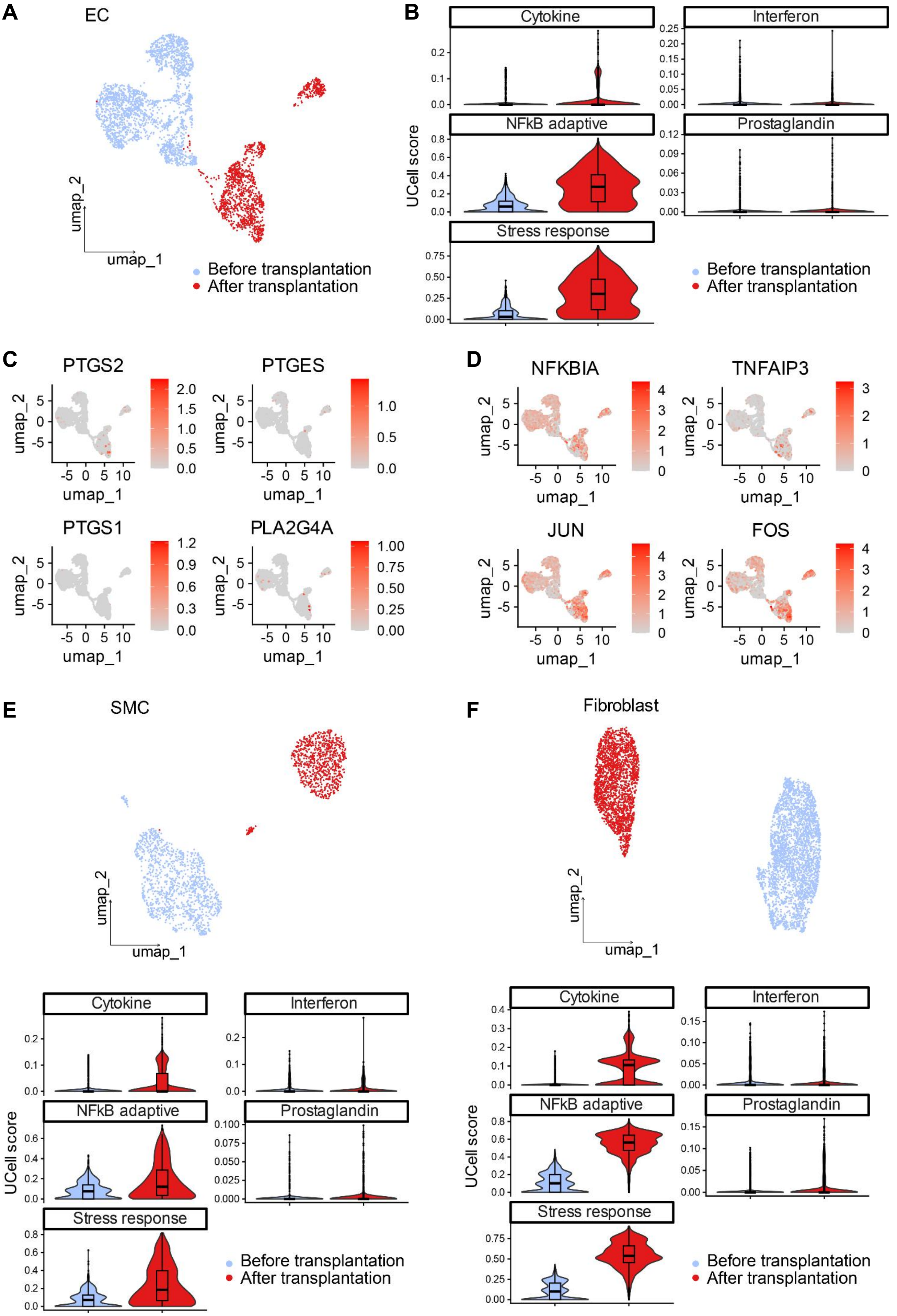

Supplement: Supplementary file 1 [file 12916_2026_5256_MOESM1_ESM.pdf]
