# Supplementary material for: 3D bioprinted human vascular organoid sheets promote functional ischemic repair and exhibit adaptive in vivo remodeling
Source: BMC Med. 2026 Sep 25;24:524. doi: 10.1186/s12916-026-05256-2 (PMC13621672; doi:10.1186/s12916-026-05256-2)
Supplement: Supplementary file 2 [file 12916_2026_5256_MOESM6_ESM.pdf]

Supplementary Figure Legend

Figure S1. Characterization of the GelMA+Matrigel Hydrogel.

A) Dependence of the complex viscosity of different hydrogels（G: 5% GelMA, G-M: 5% GelMA + 10% Matrigel）at increasing temperatures. B) The viscosity decreases with the shear rate at 19 °C. C) Effect of shear strain on storage modulus (G′) and loss modulus (G″) at 19 ℃. D) Effect of temperature on storage modulus (G′) and loss modulus (G″). E) Cell viability of hPSCs encapsulated within 3D bioprinted constructs was evaluated by Calcein-AM/PI staining after printing. F) Cell proliferation of hPSCs cultured in different bioinks was assessed by CCK-8 assay from day 3 to day 7 (n = 3 biological replicates, two-tailed unpaired Student's t-test). G) Representative bright-field images showing the morphological changes and vascular-like structure formation of 3D bioprinted constructs containing hPSC-derived ECs and SMCs at different culture time points.

Figure S2. Optimization of cell ratio for hVOS fabrication.

A) Representative immunofluorescence images of CD31-positive vascular structures formed by hVOS constructs with different EC:SMC ratios. B) Quantification of vascular network parameters, including sum tube area, sum tube length, and number of tube junction (n = 3 imaging fields, one-way ANOVA test). C) Representative immunofluorescence staining of 3D-bioprinted HUVECs + hiPSC-SMCs. D) Related quantitative analysis of sum tube area, sum tube length, and number of tube junction (n = 3 biological replicates, two-tailed unpaired Student's t-test).

Figure S3. GO and comparative analysis of cell populations in bioprinted hVOS.

A) Enriched GO terms results of EC cluster. B) Enriched GO terms results of the SMC cluster. C) Enriched GO terms results of Fibroblast cluster. D) Enriched GO terms results of the Macrophage cluster. E) UMAP visualization of the scRNA-seq dataset from the 3D bioprinted hVOS ECs with vivo vascular cells. F) UMAP visualization of the scRNA-seq dataset from the 3D bioprinted hVOS SMCs with vivo vascular cells.

Figure S4. Transcriptomic comparison of day-7 and day-14 hVOS ECs with 2D-cultured ECs.

A) Dot plot showing the expression of representative genes associated with endothelial cell identity, vascular organization, mechanotransduction, hypoxic adaptation, and proliferation in day-7 hVOS ECs, day-14 hVOS ECs, and 2D-cultured ECs. dot size indicates the percentage of cells within each cluster expressing the gene. B) Representative Gene Ontology biological processes enriched among genes upregulated and downregulated in day-7 hVOS ECs relative to 2D ECs. Bar length represents the number of genes associated with each enriched term, and bar color indicates the adjusted P value. C) Representative Gene Ontology biological processes enriched among genes upregulated and downregulated in day-14 hVOS ECs relative to 2D ECs. Bar length represents the number of genes associated with each enriched term, and bar color indicates the adjusted P value.

Figure S5. Transcriptomic comparison of day-7 and day-14 hVOS SMCs with 2D-cultured SMCs.

A) Dot plot showing the expression of representative genes associated with smooth muscle cell identity, contractile maturation, mechanotransduction, extracellular matrix remodeling, hypoxic adaptation, and proliferation in day-7 hVOS SMCs, day-14 hVOS SMCs, and 2D-cultured SMCs. Dot size represents the percentage of cells expressing each gene, and color indicates the scaled average expression level. B) Representative Gene Ontology biological processes enriched among genes upregulated or downregulated in day-7 hVOS SMCs relative to 2D SMCs. Bar length represents the number of genes associated with each enriched term, and bar color indicates the adjusted P value. C) Representative Gene Ontology biological processes enriched among genes upregulated or downregulated in day-14 hVOS SMCs relative to 2D SMCs. Bar length represents the number of genes associated with each enriched term, and bar color indicates the adjusted P value.

Figure S6. Images of the hindlimb from sham and HLI mice.

The images previously displayed in the main figure were no longer shown.

Figure S7. Images of the GM bioink and 3D bioprinted hVOS transplanted mice.

The images previously displayed in the main figure were no longer shown.

Figure S8. Transplanted hVOS reconstructs tissue architecture and forms functional human vasculature in ischemic hindlimb.

A) Representative H&E staining of sham, hVOS engraftment groups on 7, 14, and 28 days post-transplantation. B-C) Representative staining of hCD31(red) and hKu80 (green) of the above series. D) Quantitative analysis of vessel density (n = 3 biological replicates, one-way ANOVA test). E) Quantitative analysis of hKu80 positive cells (n = 3 biological replicates, one-way ANOVA test). P-values are as indicated.

Figure S9. ScRNA-seq analysis of different cell types in hVOS following transplantation.

A) Pie chart illustrating the percentage distribution of each cell population in transplanted hVOS. B) Dot plot showing expression of cluster marker genes. Dot color represents the scaled average expression level; dot size indicates the percentage of cells within each cluster expressing the gene. C) Dot plot showing expression of canonical marker genes used to define EC subtypes. Dot color represents the scaled average expression level; dot size indicates the percentage of cells within each cluster expressing the gene. D) Enriched GO terms of activated EC. E) UMAP visualization of the EC populations from the transplanted hVOS and transplanted BVO. F) Dot plot showing expression of artery and vein genes. Dot color represents the scaled average expression level; dot size indicates the percentage of cells within each cluster expressing the gene. G) UMAP visualization of the SMC populations from the transplanted hVOS and hVOS in vitro. H) Bar plot of significantly enriched GO terms for genes upregulated in SMC populations from the transplanted hVOS. I) UMAP visualization of the fibroblast populations from the transplanted hVOS and hVOS in vitro. J) Bar plot of significantly enriched GO terms for genes upregulated in fibroblast populations from the transplanted hVOS.

Figure S10. Transplantation induces a venous-biased endothelial state transition.

UMAP visualization of ECs before and after transplantation. (B) UCell scores of arterial, venous, capillary,

angiogenic, inflammatory, and cell-cycle signatures. (C) Feature plots of representative arterial- and venous-associated markers. (D) Flow cytometry analysis of pan-endothelial marker CD31, the arterial-associated marker CXCR4 and the venous-associated marker NT5E in ECs before and after transplantation.

Figure S11. Transcriptional profiling reveals inflammatory and stress-response signatures in transplanted endothelial and stromal populations.

A) UMAP visualization of ECs before and after transplantation. B) UCell-based analysis of inflammatory and stress-related signatures in ECs. C) Feature plots showing prostaglandin-associated genes (PTGS2, PTGES, PTGS1, and PLA2G4A) in ECs before and after transplantation. D) Feature plots showing NF-κB-associated genes (NFKBIA, TNFAIP3, JUN, and FOS) in ECs before and after transplantation. E) UMAP visualization and UCell analysis of SMCs before and after transplantation. F) UMAP visualization and UCell analysis of fibroblasts before and after transplantation.

Supplementary Video 1. Intravital two-photon imaging of GFP-labeled hVOS at day 14 after transplantation.

GFP-positive hVOS-derived cells are shown in green, whereas perfused blood vessels labeled by Texas Red–dextran are shown in red.

Supplementary Video 2. Intravital two-photon imaging of GFP-labeled hVOS at day 28 after transplantation.

GFP-positive hVOS-derived cells are shown in green, whereas perfused blood vessels labeled by Texas Red–dextran are shown in red.

Additional file 1: Figures S1-S11.

Figure S1- Characterization of the GelMA+Matrigel Hydrogel. Figure S2- Optimization of cell ratio for hVOS fabrication. Figure S3- GO and comparative analysis of cell populations in bioprinted hVOS.

Figure S4- Transcriptomic comparison of day-7 and day-14 hVOS ECs with 2D-cultured ECs. Figure S5- Transcriptomic comparison of day-7 and day-14 hVOS SMCs with 2D-cultured SMCs. Figure S6- Images of the hindlimb from sham and HLI mice. Figure S7- Images of the GM bioink and 3D bioprinted hVOS transplanted mice. Figure S8- Transplanted hVOS reconstructs tissue architecture and forms functional human vasculature in ischemic hindlimb. Figure S9- ScRNA-seq analysis of different cell types in hVOS following transplantation. Figure S10- Transplantation induces a venous-biased endothelial state transition. Figure S11- Transcriptional profiling reveals inflammatory and stress-response signatures in transplanted endothelial and stromal populations.

Additional file 2: Supplementary Video 1.

Supplementary Video 1- Intravital two-photon imaging of GFP-labeled hVOS at day 14 after transplantation.

Additional file 3: Supplementary Video 2.

Supplementary Video 2- Intravital two-photon imaging of GFP-labeled hVOS at day 28 after transplantation.

Additional file 4: Supplementary Table 1.

Supplementary Table 1- Gene signatures used for UCell scoring of endothelial states.

Additional file 5: Supplementary Table 2.

Supplementary Table 2- Gene signatures used for UCell scoring of inflammatory and stress-related transcriptional programs.
